# Surgical procedures and postsurgical tissue processing significantly affect expression of genes and EGFR-pathway proteins in colorectal cancer tissue

**DOI:** 10.18632/oncotarget.2669

**Published:** 2014-11-03

**Authors:** Kerstin A. David, Florian T. Unger, Philipp Uhlig, Hartmut Juhl, Helen M. Moore, Carolyn Compton, Björn Nashan, Arnulf Dörner, Andreas de Weerth, Carsten Zornig

**Affiliations:** ^1^ Indivumed GmbH, Hamburg, Germany; ^2^ Biorepositories and Biospecimen Research Branch, National Cancer Institute, National Institutes of Health, Bethesda, MD; ^3^ Arizona State University, Phoenix, AZ; ^4^ Clinic for Hepatobiliary Surgery and Transplantation Surgery, University Hospital Hamburg Eppendorf, Hamburg, Germany; ^5^ Clinic for General and Visceral Surgery and Clinic for Gastroenterology, Agaplesion Diakonieklinikum Hamburg, Hamburg, Germany; ^6^ Surgical Clinic, Israelitisches Krankenhaus in Hamburg, Hamburg, Germany

**Keywords:** Biospecimen, predictive biomarker, drug development, tissue quality, EGFR pathway, phosphoproteins

## Abstract

An understanding of tissue data variability in relation to processing techniques during and postsurgery would be desirable when testing surgical specimens for clinical diagnostics, drug development, or identification of predictive biomarkers.

Specimens of normal and colorectal cancer (CRC) tissues removed during colon and liver resection surgery were obtained at the beginning of surgery and postsurgically, tissue was fixed at 10, 20, and 45 minutes. Specimens were analyzed from 50 patients with primary CRC and 43 with intrahepatic metastasis of CRC using a whole genome gene expression array. Additionally, we focused on the epidermal growth factor receptor pathway and quantified proteins and their phosphorylation status in relation to tissue processing timepoints.

Gene and protein expression data obtained from colorectal and liver specimens were influenced by tissue handling during surgery and by postsurgical processing time. To obtain reliable expression data, tissue processing for research and diagnostic purposes needs to be highly standardized.

## INTRODUCTION

The development of personalized medicine in oncology (determining an individual's disease risk, prognosis, and therapeutic options) is fostered by high-throughput analysis of molecular biomarkers in human cancer biospecimens [1]. Insufficient quality of such specimens may lead to spurious results and data misinterpretation [2]. Biospecimen quality depends on the pre-analytical conditions in which it was acquired [3]. Of critical importance is the time interval between reducing blood supply and removing the tissue (warm ischemia time), and the time interval between removing the tissue and preserving its molecular composition (cold ischemia time) [4-8]. In addition, patients (cells) are exposed to drugs and/or are manipulated in ways that may influence expression profiles and pathway activity, resulting in inaccurate analytical data. However, there are no systematic studies analyzing the impact of pre-analytical factors.

The present study was conducted to gain a better understanding of the effects that warm and cold ischemia have on the molecular composition of a tissue specimen.

Specimens from normal and colorectal cancer (CRC) tissue, normal liver and intrahepatic metastases of CRC were collected in a highly standardized manner by specially trained staff present during entire surgery, both before surgery (presurgery), after hepatic pedicle clamping (post-clamping), and at 10, 20, and 45 minutes after resection of the tumor (10', 20', and 45' postsurgery, respectively). The molecular composition was investigated on the RNA, protein, and protein phosphorylation levels comparing normal tissue (with a rather homogeneous cell composition) with cancer tissue (representing a highly heterogeneous tissue with different ratios of malignant and [varying subtypes] of non-malignant cells).

The ultimate goals of this study were to:

- Use whole genome gene expression analysis to provide a list of genes whose expression was unstable under warm and cold ischemic conditions and would need to be analyzed with caution in research and development programs.
- Determine variability of epidermal growth factor receptor (EGFR)-pathway proteins and their phosphorylation status with respect to tissue processing as examples of critical clinical biomarkers whose expression and activity level inform targeted therapy evaluation in cancer.
- Identify genes and proteins whose expression significantly changes during and after surgery, and therefore may serve as biomarkers of tissue quality.

## RESULTS

### Patient recruitment

Fifty patients with primary CRC and 43 with intrahepatic metastasis of CRC were enrolled in the study. From the 50 patients with a primary tumor, 370 formalin-fixed paraffin-embedded (FFPE) and 780 frozen in liquid nitrogen (FF) tissue samples were collected, and from the 43 patients with metastasized cancer, 592 FFPE and 642 FF tissue samples were collected. All samples were subjected to morphological quality control (Supplementary Figure 1).

### Surgery and postsurgical tissue processing significantly affects gene expression in normal colon, normal liver, and CRC tissue

**Figure 1:**
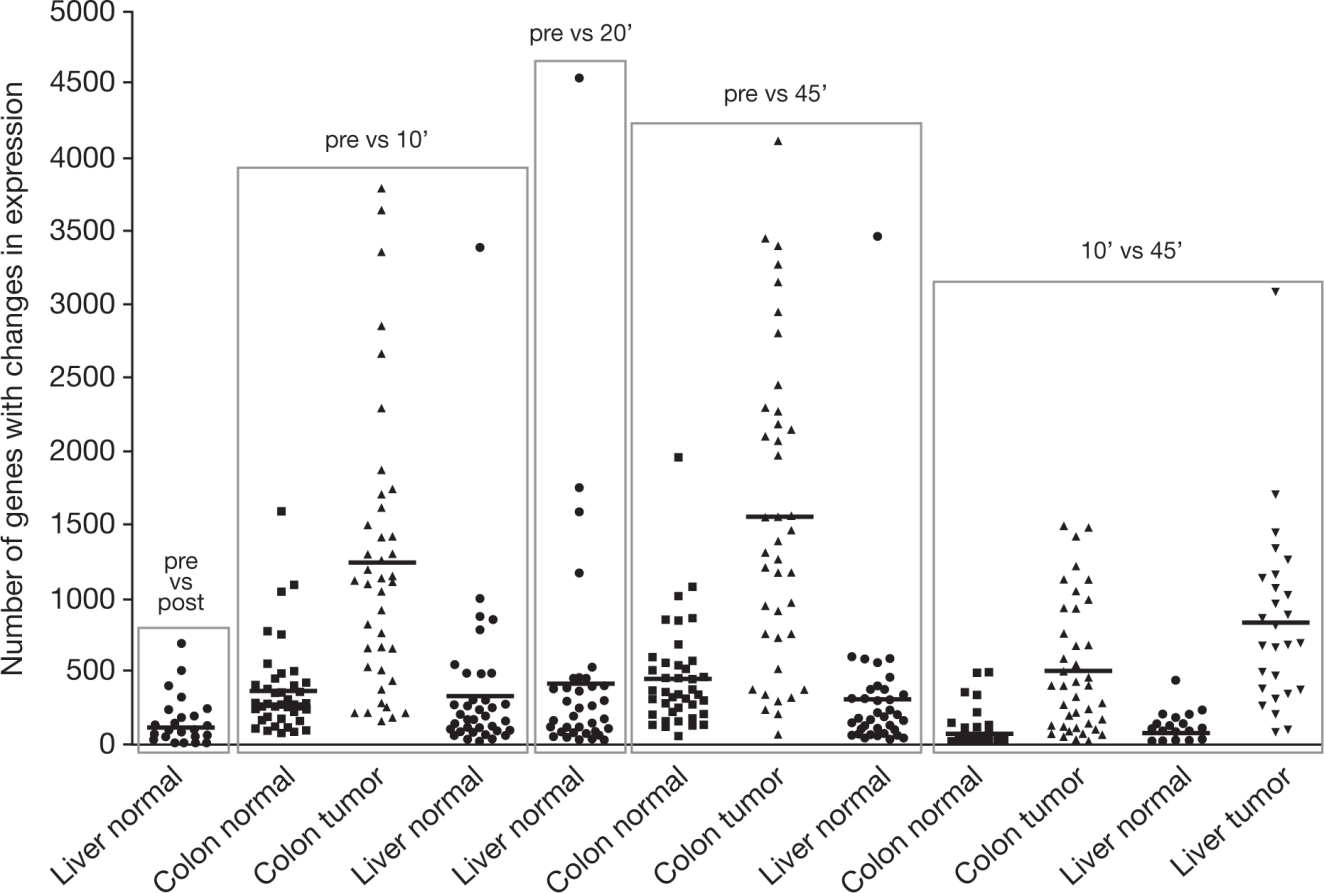
The variability of gene expression changes between patients, tissue type, surgery, and tissue processing times The figure shows the number of genes whose expression changed by more than 2-fold according to the tissue source and timing of pedicle clamping and postsurgical processing: pre, before hepatic pedicle clamping; post, after clamping; 10', 10 minutes after resection; 20', 20 minutes after resection; and 45', 45 minutes after resection. Bars represent mean numbers of gene expression changes.

Ten-minute clamping time of the hepatic artery significantly changed the expression of up to 690 (mean 118) genes in normal liver. The number of affected genes increased with surgery time (Figure 1). While some genes normalized (vs first biopsy), other genes had significant changes in expression level with prolonged surgery and postsurgical tissue processing time. The number of affected genes in normal liver and normal colon was similar (Table 1). In contrast to normal tissue, the variability of gene expression in relation to surgery and postsurgical processing time was significantly higher in cancer tissue. Within 10' and 45' postsurgery, up to 3,087 (mean 830) genes in metastatic liver CRC tumors showed ≥2-fold and significant difference in expression. In primary CRC tissue, comparison between presurgery and 10' postsurgery biopsies identified up to 3,792 (mean 1,234) genes and 45' postsurgery biopsies identified up to 4,116 (mean 1,553) genes. Supplementary Tables 1–4 summarize all genes that showed a significant and ≥2-fold change in expression during surgery and postsurgical processing.

**Table 1:** Differentially expressed genes in normal colon and liver tissues Normal colon tissue is shown above the line and normal liver tissue shown below the line. Expression recorded: pre, before hepatic pedicle clamping; post, after clamping; 10', 10 minutes after resection; and 45', 45 minutes after resection. *p<0.05

Probe ID	Gene	Protein	Pre vs 10'	Pre vs 45'
1555827_at	*CCNL1*	Cyclin L1	*	*
201324_at	*EMP1*	Epithelial membrane protein 1	*	*
201325_s_at			*	
202499_s_at	*SLC2A3*	Solute carrier family 2 (facilitated glucose transporter), member 3	*	*
202672_s_at	*ATF3*	Activating transcription factor 3	*	
202988_s_at	*RGS1*	Regulator of G-protein signaling 1	*	*
216834_at			*	*
215034_s_at	*TM4SF1*	Transmembrane 4 L six family member 1	*	*
227697_at	*SOCS3*	Suppressor of cytokine signaling 3	*	*
232304_at	*PELI1*	Pellino homolog 1 (Drosophila)	*	*
36711_at	*MAFF*	V-maf musculoaponeurotic fibrosarcoma oncogene homolog F (avian)	*	
202291_s_at	*MGP*	Matrix GLA protein		*
209101_at	*CTGF*	Connective tissue growth factor		*
228335_at	*CLDN11*	Claudin 11		*
202859_x_at	*IL8*	Interleukin 8		*
228528_at	*LOC100286909*	Hypothetical protein LOC100286909		*

### Identification of potential RNA-based tissue quality biomarkers

Hierarchical clustering was used to further evaluate gene expression data and to categorize different patient groups. Clustering of data from patients who had colon surgery (normal and tumor tissue clustering separately) resulted in seven different partitions. Most patients (89% with normal tissue) fell into the partition (presurgery/10', 20', 45' postsurgery) meaning that the presurgery timepoint separated from the 10', 20', and 45' postsurgery timepoints.

Excluding five patients who were regarded as outliers, only patients following the partition presurgery/10' 20' 45' postsurgery in normal tissue were used to compare gene expression intensity levels with multiple t-tests between the presurgery and 10' postsurgery timepoints, and presurgery and 45' postsurgery timepoints. In normal colon tissue, 70 probes showed a differential expression in both comparisons. Of these, seven probes (encoding for five different genes) had a log-fold change of ≥2 in both comparisons (Table 2). Expression of another five genes showed ≥2 log-fold change in the comparison presurgery versus 45' postsurgery only, while the log-fold change was slightly lower in the presurgery versus 10' postsurgery comparison. Genes that were significantly up-regulated upon resection of normal colon tissue comprised transcription factors (*EGR1*, *FOS*), signaling molecules (*CYR61*, *RGS1*, *SGK1*) of the extracellular matrix, and dual specificity phosphatase 1 (*DUSP1*, a protein that dephosphorylates MAPK1). Gene expression for mucosal proteins such as dual oxidase 2 (*DUOX2*, a protein that plays a role in antimicrobial defense), solute carrier family 6 (*SLC6A14*, a protein mediating amino acid transport), and vanin 1 (*VNN1*, a protein involved in vitamin B5 recycling), was down-regulated. In colon tumor tissue, similar gene expression changes were found for *CYR61*, *RGS1*, *DUSP1*, *DUOX2*, and *SLC6A14*, although the log-fold change was generally lower compared to normal tissue (Table 2).

The same approach was not applicable to normal liver tissue because of the low number of affected genes and a more diverse change of expression (data not shown).

**Table 2:** Differentially expressed genes in normal and colorectal tumor tissue. Gene expression was compared: pre, before hepatic pedicle clamping; post, after clamping; 10', 10 minutes after resection; 20', 20 minutes after resection; and 45', 45 minutes after resection

			Normal colon tissue				Colorectal tumor tissue			
			Pre vs 10'		Pre vs 45'		Pre vs 10'		Pre v. 45'	
Probe ID	Gene	Protein and function	p-value	Log-fold change	p-value	Log-fold change	p-value	Log-fold change	p-value	Log-fold change
201289_at	*CYR61*	Cysteine-rich angiogenic inducer 61; extracellular matrix-associated signaling protein that plays important roles in tissue repair	1.57E-18	**2.49**	2.84E-22	**2.55**	0.00000133	**1.41**	3.96E-08	**1.59**
202988_s_at	*RGS1*	Regulator of G-protein signaling 1; attenuates signaling activity of G-proteins	4.21E-21	**2.34**	5.05E-25	**2.39**	1.81E-09	**1.57**	7.62E-12	**1.91**
216834_at			1.65E-22	**2.33**	8.96E-25	**2.36**	3.06E-09	**1.42**	5.2E-12	**1.76**
201694_s_at	*EGR1*	Early growth response 1; transcription factor	1.92E-12	**2.02**	1.79E-18	**2.26**	.	**.**	.	.
227404_s_at			1.4E-10	**1.98**	2.57E-16	**2.32**	.	**.**	.	.
201739_at	*SGK1*	Serum/glucocorticoid regulated kinase 1; activates potassium, sodium and chloride channels	4.03E-14	**2.09**	2.74E-16	**2.21**	.	**.**	.	.
209189_at	*FOS*	FBJ murine osteosarcoma viral oncogene homolog; transcription factor involved in cell proliferation, differentiation, survival, hypoxia and angiogenesis	1.26E-18	**2.77**	9.3E-25	**3.19**	.	**.**	.	.
218541_s_at	*C8orf4*	Chromosome 8 open reading frame 4; uncharacterized protein	2.39E-12	**2**	3.64E-15	**2.01**	.	**.**	.	.
201041_s_at	*DUSP1*	Dual specificity phosphatase 1; dephosphorylates MAP kinase MAPK1/ERK2	1.21E-17	**1.85**	3E-23	**2.15**	.	**.**	0.0000115	**1.14**
219727_at	*DUOX2*	Dual oxidase 2; plays a role in antimicrobial defense at the mucosal surface	3.36E-08	**-1.88**	2.24E-10	**-2.14**	0.000497	**-1.51**	0.0000133	**-1.82**
219795_at	*SLC6A14*	Solute carrier family 6 (amino acid transporter), member 14; mediates the uptake of a broad range of amino acids	0.0000101	**-1.87**	0.00000036	**-2.16**	0.0109	**-1.2**	0.000316	**-1.75**
205844_at	*VNN1*	Vanin 1; amidohydrolase recycling pantothenic acid (vitamin B5) and releasing cysteamine	0.000102	**-1.62**	0.000000164	**-2.17**	.	.	.	.

### Identification of housekeeping genes not affected by surgery and tissue processing

Gene expression data were sorted according to the coefficients of variance (CV). The 10 probe sets with the lowest CV across all four timepoints in normal tissue are listed in Table 3. While some probe sets encoded for insufficiently explored proteins, among those very constitutively-expressed genes was eukaryotic translation elongation factor 1 alpha 1 (*EEF1A1*), a widely expressed gene with high copy numbers that is known as a potential housekeeping gene for gene expression analysis [9]. Further well-known housekeeping genes with low CV were ribosomal proteins L13 and S18, beta-glucuronidase, and beta-actin, while other frequently used housekeeping genes, such as beta-2-microglobulin and beta2B-tubulin, were not constitutively expressed.

The same exercise was conducted for gene expression in CRC tissue (data not shown). Again, *EEF1A1* expression showed a very low CV, suggesting it may function as a reference gene in both normal and neoplastic colorectal tissue.

**Table 3:** The 10 probe sets (9 genes) with the lowest coefficient of variation (CV) across all four timepoints (presurgery and 10, 20, and 45 minutes after resection) in normal colon tissue are shown above the dotted line, and 20 well-known housekeeping genes (HKG) with their CV and their rank when sorted for CV are shown below the dotted line

			Normal tissue		Tumor tissue
Probe ID	Gene	Protein and function	CV	Rank	Rank
1558623_at	*LOC729121*	Hypothetical LOC729121	0.0000651769	1	22,547
227472_at	*DDA1*	DET1 and DDB1 associated 1; may be involved in ubiquitination and subsequent proteosomal degradation	0.0000763301	2	5,095
213477_x_at	*EEF1A1*	Eukaryotic translation elongation factor 1 alpha 1; involved in protein biosynthesis	0.000120035	3	70
206559_x_at			0.00021194	10	464
203172_at	*FXR2*	Fragile X mental retardation, autosomal homolog 2; RNA-binding protein	0.00012495	4	5,833
202652_at	*APBB1*	Amyloid beta (A4) precursor protein-binding, family B, member 1; transcription coregulator	0.000140598	5	22,323
209394_at	*ASMTL*	Acetylserotonin O-methyltransferase-like; unknown function	0.000153558	6	28,176
234891_at	*DKFZP547L112*	Hypothetical protein DKFZp547L112	0.000198356	7	17,884
212986_s_at	*TLK2*	Tousled-like kinase 2; involved in chromatin assembly	0.000208644	8	2,237
223148_at	*PIGS*	Phosphatidylinositol glycan anchor biosynthesis, class S; component of the GPI transamidase complex	0.000210952	9	24,752
210646_x_at	*RPL13A*	Ribosomal protein L13a, HKG	0.000370481	53	752
201049_s_at	*RPS18*	Ribosomal protein S18, HKG	0.000581215	185	2,319
230125_at	*GUSB*	Glucuronidase, beta, HKG	0.000939722	647	19,340
AFFX-HSAC07/X00351_3_at	*ACTB*	Actin, beta, HKG	0.001080551	941	7,550
216457_s_at	*SF3A1*	Splicing factor 3a, subunit 1, 120 kDa, HKG	0.001542067	2,363	890
212581_x_at	*GAPDH*	Glyceraldehyde-3-phosphate dehydrogenase, HKG	0.001735025	3,208	332
201093_x_at	*SDHA*	Succinate dehydrogenase complex, subunit A, flavoprotein (Fp), HKG	0.002533378	8,062	42,630
203135_at	*TBP*	TATA box binding protein, HKG	0.003036083	12,044	11,750
203040_s_at	*HMBS*	Hydroxymethylbilane synthase, HKG	0.003145361	12,988	6,489
1565446_at	*HPRT1*	Hypoxanthine phosphoribosyltransferase 1, HKG	0.003323065	14,616	23,933
229165_at	*MRPL12*	Mitochondrial ribosomal protein L12, HKG	0.003428532	15,543	12,742
224695_at	*C2orf29*	Chromosome 2 open reading frame 29, HKG	0.00344027	15,651	6,081
211296_x_at	*UBC*	Ubiquitin C, HKG	0.003508935	16,259	2,040
235741_at	*PPIA*	Peptidylprolyl isomerase A (cyclophilin A), HKG	0.003666547	17,628	33,077
221842_s_at	*ZNF131*	Zinc finger protein 131, HKG	0.003960494	20,273	20,072
227708_at	*EEF1A1*	Eukaryotic translation elongation factor 1 alpha 1, HKG	0.004407467	24,160	24,765
1566191_at	*SUZ12*	Suppressor of zeste 12 homolog (*Drosophila*), HKG	0.005051028	29,227	49,826
240686_x_at	*TFRC*	Transferrin receptor (p90, CD71), HKG	0.006000472	35,483	30,303
232311_at	*B2M*	Beta-2-microglobulin, HKG	0.012872315	49,756	44,528
214023_x_at	*TUBB2B*	Tubulin, beta 2B, HKG	0.019826709	52,506	53,778

### Up to 60% of patients showed changes of EGFR-pathway protein expression

EGFR and its downstream key signaling proteins of the AKT and MAPK pathway were investigated in relation to total protein concentration and phosphorylation status. Using expression levels of presurgery biopsies as a reference, changes (up or down) in protein expression of ≥2-fold were documented. In normal liver tissue the preclamping tissue biopsy was used as reference.

Overall, changes in the expression of EGFR-pathway proteins were lowest in normal liver, higher in normal colon, and highest in cancer tissue (Supplementary Figure 2). In liver tissue, a change in total protein expression was not observed, while in normal colon tissue EGFR expression changed ≥2-fold in 10% of patients, while expression of the downstream protein p70-S6K changed >2-fold in 35% of patients between presurgery and 45' postsurgery. In CRC tissue, expression changes in EGFR-related proteins were striking. Between presurgery and 10' postsurgery, EGFR total protein expression changed by >2-fold in 20% of all patients and in 30% of all patients within a further 35 minutes (presurgery/45' postsurgery). Subsequently, expression of downstream proteins, such as p70-S6K, changed >2-fold from presurgery to 45' postsurgery in more than 60% of patients.

**Figure 2:**
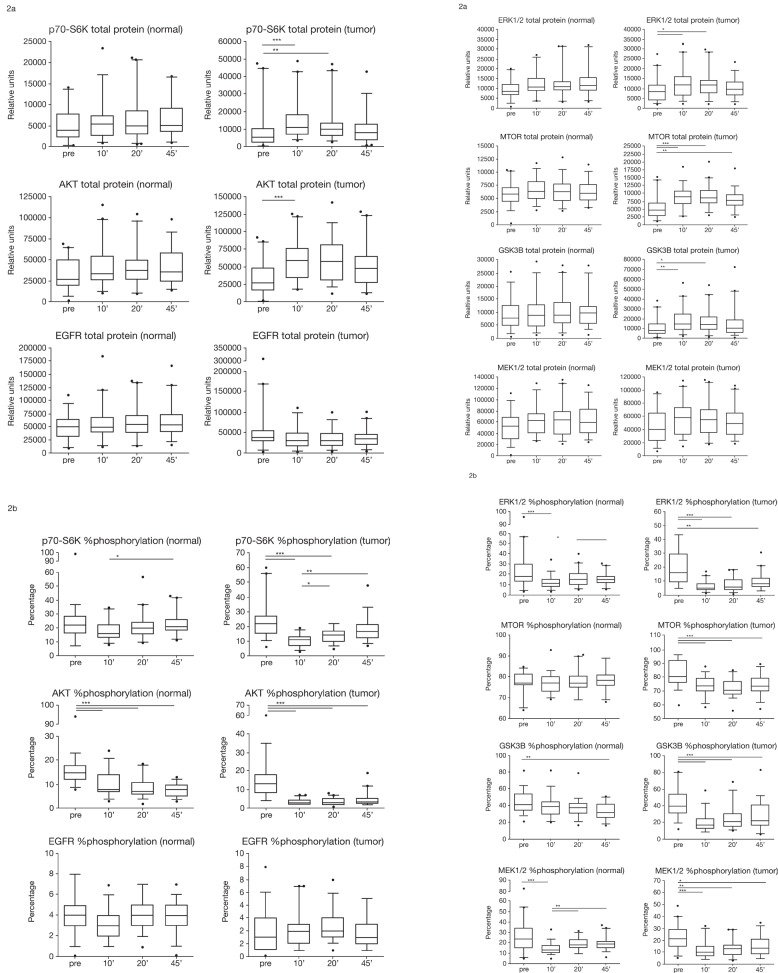
(A) Total protein expression (relative units) of p70-S6K, AKT, EGFR, ERK1/2, MTOR, GSK3B, and MEK1/2 in normal and tumor colon tissue at four timepoints of tissue collection: pre, before hepatic pedicle clamping; 10', 10 minutes after resection; 20', 20 minutes after resection; and 45', 45 minutes after resection.*p<0.05; **p<0.01; ***p<0.001. Box plots indicate the 5/95% confidence interval, median, and standard deviation. (B) Percentage of protein phosphorylation of p70-S6K, AKT, EGFR, ERK1/2, MTOR, GSK3B, and MEK1/2 in normal and tumor colon tissue at four timepoints of tissue collection: pre, before hepatic pedicle clamping; 10', 10 minutes after resection; 20', 20 minutes after resection; and 45', 45 minutes after resection. *p<0.05; **p<0.01; ***p<0.001. Box plots indicate the 5/95% confidence interval, median, and standard deviation.

While the change in total protein level became statistically significant for AKT, MTOR, ERK1/2, GSK3B, p70-S6K (Figure 2), and HIF1A (data not shown), other proteins (Figure 2) such as EGFR showed up- and down-regulation in individual patients and, while still showing unstable expression in some patients, it was not statistically significant (Supplementary Figure 3).

### Surgery and postsurgical processing strongly affects phosphorylation of key signaling molecules within the AKT and MAPK pathway

The phosphorylation status of key signaling proteins was significantly affected in most patients and to a larger extent in tumor tissue compared with normal tissue. We found a chain of phosphorylation events indicating activation in some and inactivation in other parts of the phosphorylation cascade (data not shown). While these changes remain to be evaluated by a more detailed analysis of clinical variables, we found statistically significant changes in most key regulatory proteins between presurgery and 10' postsurgery samples and additional changes for some proteins during the postsurgical cold ischemia time. This included AKT, MTOR, ERK1/2, and MEK (Figure 2).

In normal colon and liver tissue, total levels of most proteins did not differ significantly between timepoints. However, there was a statistically significant increase at 10' postsurgery for AKT and MTOR in CRC tissue. Protein phosphorylation decreased significantly with warm and cold ischemia in normal colon and CRC tumor tissue (Figure 2). It is important to note that protein phosphorylation showed similar trends for changes in selected proteins in both tissue types (normal tissue and highly heterogeneous tumor tissue). This implies that these regulations occur irrespective of the cell type and, thus, could serve as tissue quality marker independent of the kind of tissue (normal and tumor).

**Figure 3:**
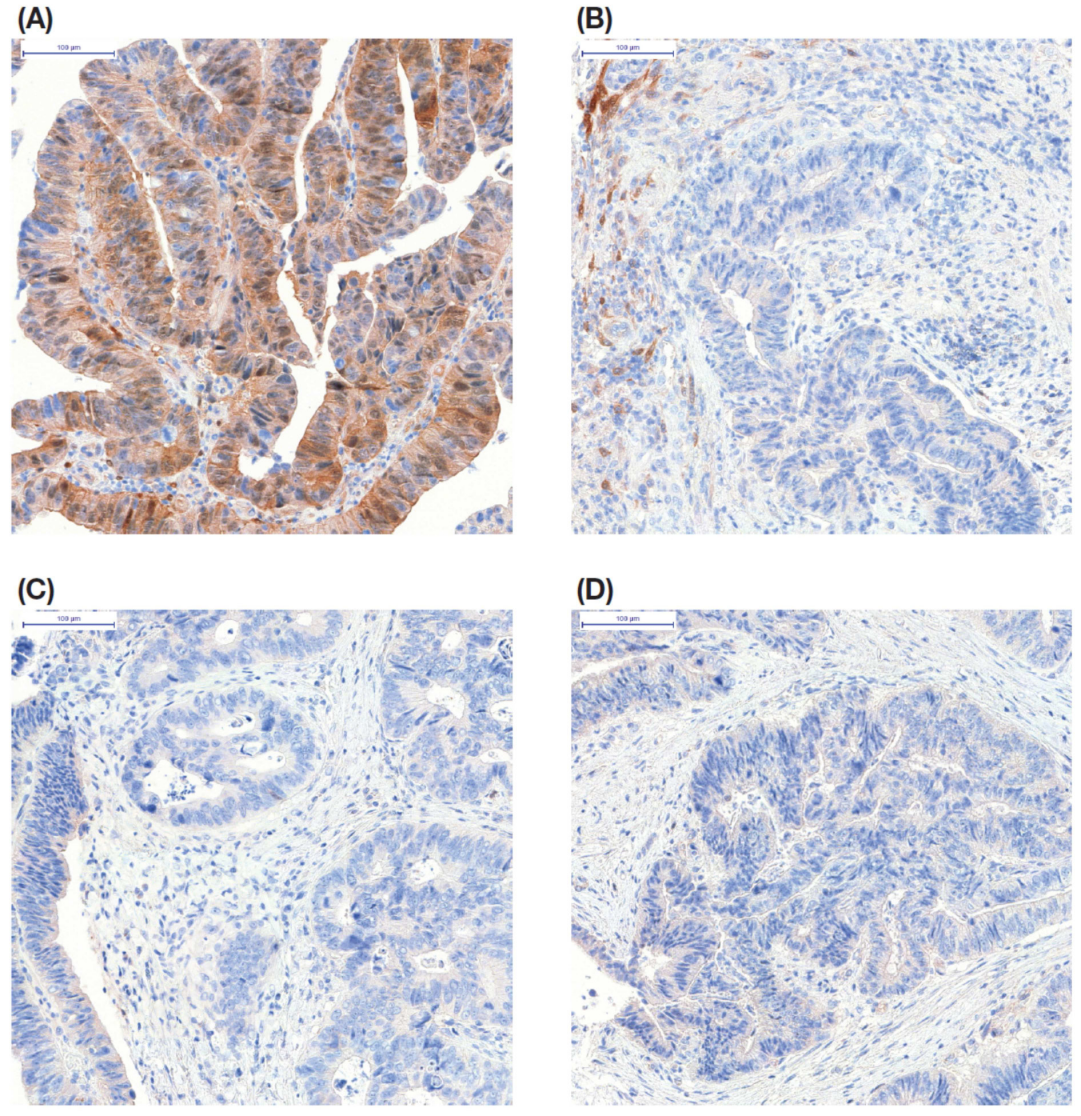
Representative immunohistochemistry for pAKT on formalin-fixed colon cancer tissue from one patient taken at four timepoints (A) biopsy presurgery; (B) tissue fixed 10 minutes after resection; (C) tissue fixed 20 minutes after resection; and (D) tissue fixed 45 minutes after resection.

The above mentioned decline in protein phosphorylation was mostly associated with decreased stain intensity upon immunohistochemistry, which was statistically significant for phosphorylated EGFR, AKT, and ERK1/2. Example images of immunohistochemistry for the detection of p-AKT in a patient with colon cancer in relation to ischemia time are shown in Figure 3.

### HSP27 phosphorylation increases with warm and cold ischemia

HSP27 was evaluated as its expression is known to respond to cellular stress [10]. While total HSP27 protein levels were similar across all groups, the percentage of phosphorylated versus total HSP27 increased over time in an almost linear fashion from presurgery to 45' postsurgery in most patients, demonstrating a statistically significant difference between presurgery/preclamping samples versus those taken after prolonged cold ischemia (Supplementary Figure 4). After 45' postsurgery of cold ischemia, the proportion of HSP27 phosphorylation had increased 8-fold compared to presurgery levels. In normal liver tissue it had increased 2-fold.

## DISCUSSION

Pharmaceutical companies put significant efforts into the development of specific pathway inhibitors, and new drugs can emerge in high numbers from preclinical development programs. Identification of drug targets as stratification and predictive biomarkers in patient populations has become an important field in drug development; indeed, such biomarkers are thought to be essential for current and future patient care and an important strategy in controlling reimbursement costs in cancer care.

While DNA aberrations such as activating mutations in specific genes allow the identification to some extent of drug targets under various circumstances, this approach is of limited predictive value. Utilization of protein targets and cancer pathway activity as determined by quantifying phosphorylation of regulatory proteins can provide a much deeper insight into a patient's individual tumor biology. However, a major challenge for the discovery and development of predictive drug targets is the need for tissue samples that truly represent the reality of a patient's tumor biology. As most tissues are surgical specimens, a major concern is the risk of modified expression levels because of tissue manipulation during and after surgery. Understanding the effects of surgical manipulation on cancer biomarkers will be an important part of the knowledge base upon which such biomarkers can be fully utilized to benefit patients.

Stemming the blood supply rapidly induces hypoxia and cellular stress and is thought to affect the molecular composition of cells and thus, the analytical data derived from these tissues. In addition, during surgery, patients (and, naturally, their cells) are exposed to various kinds of drugs given by the anesthetist.

In this study we demonstrate that human tissue is highly susceptible to surgical factors and prolonged postsurgical ischemia, and standardized handling of tissue is an important factor for subsequent analysis. Analyzing known stress and ischemia markers such as HSP27 [12] and HIF1A [13-14], we found that in normal liver tissue, HSP27 phosphorylation was significantly increased at 10 minutes after hepatic pedicle clamping and further increased throughout post-resection ischemia in a time-dependent manner. In normal colon tissue, HSP27 phosphorylation increased in an almost linear time-dependent manner between 10' and 45' postsurgery. Hence, HSP27 phosphorylation appears to be an interesting marker for pre- and post-resection tissue quality. We also found a significant increase in HIF1A protein levels 10' postsurgery of colorectal tumors which, however, declined again at later timepoints. Interestingly, influence of ischemia on protein phosphorylation has been detected independent from tissue type (normal vs cancer) and the cellular composition of tumor tissue with respect to tumor cell content and non-malignant cell subtypes such as stroma cells and inflammatory infiltrates.

In individual patients the expression of more than 4,000 genes were altered and up to 60% of patients with primary CRC showed more than a 2-fold expression change in proteins and their phosphorylation, which is in agreement with previous studies demonstrating cold ischemia-related alterations in gene and protein expression [4, 8]. In general, the impact on the molecular composition was more severe in tumor tissue compared to normal tissue, likely due to the higher activity of tumor cells compared to normal cells. The most striking changes were observed during warm ischemia and early cold ischemia (10' postsurgery); gene and protein expression changes during prolonged cold ischemia were surprisingly less prominent though still significant. Interestingly, up to 690 (mean 118) genes were already affected in individual patients by just clamping the hepatic pedicle for 10 minutes.

With regard to gene and protein expression data, we observed inter-individual variability across patients. Most of these patients, however, appeared to follow a similar pattern of gene expression changes. By only using those patients for further gene expression analyses, we were able to identify significant up-regulation of several transcription factors and signaling molecules of the extracellular matrix such as cysteine-rich angiogenic inducer 61 (CYR61, CCN1), and the regulator of G-protein signaling 1 (RGS1). CYR61 is a matrix cell-adhesion molecule. Depending on the context, it promotes cell proliferation, survival, apoptosis, or angiogenesis by binding to distinct integrins and plays an important role in wound repair [15]. RGS1 attenuates the signaling activity of G-proteins by fostering GTP hydrolysis and has various immunomodulatory functions [16]. Simultaneously, the expression of several genes from colonic enterocytes was down-regulated.

On the protein level, the variability between patients was also high. Protein levels and their phosphorylation status showed increasing and decreasing levels between different patients and timepoints. Variability was higher in tumor tissue, probably because of the highly heterogeneous cell composition, but also occurred in homogeneous normal tissue. This implies that the observed changes are to a large extent related to tissue processing and not just variability in tumor cell content. However, identification of tumor cell-specific events and cancer cell biomarker requires analysis of isolated cancer cells, which will be performed in follow-up studies.

However, the most consistent molecular change during tissue resection and post-surgical ischemia was a decrease in protein phosphorylation, observed for AKT, MTOR, p70-S6K, GSK3B, and ERK1/2 from colon tissue. Dephosphorylation of ERK1/2 is particularly interesting with regard to the fact that an up-regulated gene expression was found for dual specificity phosphatase 1 (DUSP1, MKP1). This protein can dephosphorylate MAPK (ERK) in the cell nucleus and thus attenuates MAPK signaling [17].

Even in our highly standardized tissue processing protocol, a comparison between frozen and formalin fixed tissue is difficult because the slow formalin penetration significantly prolongs the ischemia time (see Methods). However, the decline in phosphorylation status observed in frozen tissue had an influence on immunohistochemical staining for pERK, pAKT, and pEGFR in colon tissue, where staining intensity was often lower in postsurgery compared to presurgery samples. This has important consequences, since immunohistochemistry is frequently used to determine activation of signaling pathways in individual cancer specimens in order to determine personalized treatment options. The pathologist analyzing post-resection specimens needs to be aware of the alterations that can be induced by cold ischemia time and therefore needs precise information about the conditions under which the tissue was procured. Since DUSP1, CYR61, and RGS1 gene expression was up-regulated in both normal and CRC tissue upon tissue resection, these genes may represent interesting candidates for biomarkers of post-resection tissue quality. Further evaluations by RT-PCR and protein quantification; however, would be needed for their characterization and validation as quality biomarkers.

Our study also identified candidate “housekeeping” or relatively stable genes, the expression of which was not altered by tissue resection and post-surgical cold ischemia. In this regard, the *EEF1A1* gene appears to be particularly interesting. Its CV was very low across all four timepoints, both in normal colon tissue and in CRC tissue. *EEF1A1* is known to be constitutively expressed in many tissues and under various conditions and has been described as a useful housekeeping gene for gene expression analyses [18]. Further candidates for reference genes have been identified. Interestingly, genes that are frequently used as reference genes such as beta2-microglobulin or beta2-tubulin did not show constitutive expression throughout resection and post-surgical ischemia and therefore do not appear suitable as housekeeping genes under the described conditions.

The data presented herein are largely in agreement with a recent report that described fluctuations of protein levels and protein phosphorylation in human intestine tissue samples as a consequence of different ischemic conditions before preservation [8]. The authors reported that a general trend towards up- or down-regulation of proteins was not evident due to pronounced inter-individual variability. We were able to demonstrate a general trend towards up- and down-regulation of proteins and their phosphorylation status due to a higher number of samples that were investigated in our study, but confirm the occurrence of high inter-individual variability. In fact, we identified a majority of patients that responded towards tissue resection with a common pattern of gene expression changes, while some other patients responded in a completely different manner. These inter-individual differences require further investigation.

In summary, our data show a significant difference in the molecular composition of tissue specimens collected after tumor resection compared to specimens collected via colonoscopy before tumor resection. This difference is larger than the difference between various post-resection times between 10 and 45 minutes. The observed effect is either due to warm and cold ischemia, and/or the anesthesia/surgical procedure itself, and the manipulation of the tissue. The molecular changes induced during and after tumor resection are heteromorphic and do not occur in all patients. In general, kinase proteins become dephosphorylated, which may result in decreased intensity of immunohistochemical staining. The ratio between phosphorylated and total HSP27 protein has emerged as a promising marker for tissue quality, since it demonstrates an almost linear increase with prolonged cold ischemia time in all three tissue types.

This study presents an important contribution to the understanding of molecular changes that are being introduced into tissue samples during the pre-analytical phase, i.e. by the tissue collection procedure itself and the surgical procedures prior tissue collection. For the first time, we provide a full list of genes whose expression is altered due to tissue processing and surgical manipulation. In addition, our findings suggest that the analysis of regulatory pathway proteins and specific growth factor receptors, such as EGFR, requires the use of highly standardized and rapid tissue processing techniques. Once standardized techniques have been validated, analysis of the expression of regulatory pathway proteins may be valuable as predictive markers of targeted therapies.

## ONLINE METHODS

### Patient enrollment

Fifty patients with colorectal cancer (CRC) and 43 with hepatic metastasis of CRC who were scheduled for tumor resection surgery gave informed consent to be enrolled in the study. Only patients with a tumor larger than 3 cm in diameter were enrolled. Patients who had received chemotherapy or radiation therapy <3 weeks before surgery were excluded. Specially trained study nurses were present during all surgeries. They performed tissue processing in the surgical unit and clinical data documentation to assure that the same standardized operating procedures have been applied to all patients. The study was conducted at three sites in Hamburg, Germany, and received approval by the competent ethics review committee of the medical association Hamburg under reference no. PV3342.

### Tissue collection before and during surgery

After induction of anesthesia, patients with primary CRC underwent a colonoscopy, upon which three biopsies were taken from the tumor and three biopsies from the adjacent normal tissue (presurgery) (Supplementary Figure 1). For patients with hepatic metastasis of CRC, four pieces of tissue were taken from normal liver parenchyma before the start of liver resection, i.e. just before clamping of the hepatic artery (presurgery). About 10 minutes after hepatic pedicle clamping (post-clamping), another four tissue samples were collected from the normal liver parenchyma (postsurgery).

For all patients, 12 tissue samples were each collected from the tumor tissue and the adjacent normal tissue after resection of the tumor and adjacent normal tissue. These 24 samples were divided into three groups, each exposed to a cold ischemia time of 10 minutes (10'), 20 minutes (20'), and 45 minutes (45'), respectively. To minimize variation because of formalin penetration time (approximately 1 mm/hour), every tissue sample had a similar size of approximately 5 × 5 × 5 mm and an approximate weight of 120 mg. Since formalin penetration is slow, samples were all fixed for 16 hours. For each timepoint and tissue type (normal or tumor), half of the tissues were immediately stored in the vapor phase of liquid nitrogen, while the other half was immersion fixed in 4% buffered formaldehyde (Supplementary Figure 1).

### Processing of tissue specimens

All tissue specimens in formaldehyde were immersion fixed for 16 to 72 hours. Thereafter, they were weighed and placed in 70% ethanol for a maximum of 24 hours until further processing. Processing was conducted with an automated system (Microm tissue processor STP 420 D Thermo Scientific, Dreieich, Germany) resulting in the embedding of tissues in paraffin (Paraplast).

From each formalin-fixed paraffin-embedded (FFPE) and frozen in liquid nitrogen (FF) tissue specimen, one section was stained with hematoxylin-eosin and evaluated under a light microscope in order to verify the presence of tumor and normal tissue, respectively. Tumor content was 10–90% in tumor samples and 0% in all adjacent normal samples.

After histological quality control, FFPE and FF samples were selected for the following molecular analyses: i) quantification of total and phosphorylated protein by a medium-throughput enzyme-linked immunosorbent assay technology (Meso Scale Discovery [MSD]); ii) semi-quantitative evaluation of protein expression by immunohistochemistry; and iii) gene expression profiling on total RNA extracts using an Affymetrix whole genome chip (Supplementary Figure 1).

### Quantification of proteins

Forty FF specimens were used. Tissue lysates were prepared by cutting and homogenizing a 20 μm slice from each FF specimen. The resulting tissue lysate was subjected to a bicinchoninic acid protein assay (BCA kit; Sigma, Steinheim, Germany) to determine protein concentration. Quantification of proteins was conducted using 96-well plates with capture antibodies based on the assay platform from MSD (Gaithersburg, MD, USA). The following assay kits were used: HIF1alpha singleplex, HSP27/pHSP27(Ser15) duplex, EGFR/pEGFR(Tyr1173) duplex, AKT/pAKT(Ser473) duplex, MTOR/pMTOR(Ser2448) duplex, p70-S6K/pp70-S6K(Thr421, Ser424) duplex, GSK3B/pGSK3B(Ser9) duplex, MEK1/2/pMEK1/2(Ser217/221) duplex and ERK1/2/pERK1/2(Thr202/Tyr204, Thr185/Tyr187) duplex.

Assays were performed using 10 μg of tissue lysate according to the manufacturers' instructions and analyzed with the SECTOR Imager platform (MSD). Analyses were conducted in triplicate and arithmetic mean values were calculated. Mean values of post-surgery samples were against presurgery samples from the same patient. The percentage of phosphorylation was calculated according to the formula: phosphorylation (%) = (DFx phosphorylated protein)/(phosphorylated protein + total protein) x 100 with distribution factor (DF) = 2. If the phosphorylation (%) was >100, DF was adjusted. In order to account for inter-assay variation, lysates of stimulated human cells were produced and employed as positive and negative controls.

### Immunohistochemistry

5 μm sections from FFPE tissue were mounted on glass slides, air dried at 56°C overnight and subjected to immunostaining using an automated platform (Ventana Discovery XT, Tucson, AZ, USA). The following primary antibodies and dilutions were used: pERK1/2 1:300, pAkt(Ser473) 1:30, pEGFR 1:110, pmTOR 1:130, pHer3 1:70 (all from Cell Signaling Technology Inc., Danvers, MA, USA). After staining, sections were treated with ascending ethanol concentrations and xylene and were finally covered with Pertex (Medite GmbH, Burgdorf, Germany). Sections were examined under a light microscope by a pathologist. Tumor cell staining was classified as absent, weak, moderate, or strong and a staining score was calculated based on the extent of staining according to the formula: score = 3 × percentage of strongly stained tumor cells + 2 × percentage of moderately stained tumor cells + 1 × percentage of weakly stained tumor cells. All IHC staining protocols used within this study are “fit for purpose” and validated according to FDA guidelines, and staining procedures were also conducted according to standard operating procedures. Therefore, assays are controlled in terms of specificity and accuracy.

### Gene expression analysis

For gene expression analysis, total RNA was extracted in duplicates from every frozen tissue block. Briefly, tissues were homogenized and RNA was isolated in two steps using phenol chloroform extraction and the RNeasy MinElute Cleanup Kit (Qiagen, Hilden, Germany) according to the manufacturer's instructions. RNA quality was evaluated based on 18S and 28S ribosomal RNA peaks using the Agilent 2100 bioanalyzer (Agilent Technologies, Berlin, Germany). Only RNA samples with an RNA integrity number >7 were used for gene expression analysis. RNA samples were analyzed in replicates using oligonucleotide microarrays (GeneChip Human Genome U133 Plus 2.0) based on the Affymetrix GeneChip™ technology (Affymetrix Inc., Santa Clara, CA, USA).

### Statistical analysis

Statistical analysis of protein content measured on the MSD platform and statistical analysis of staining scores derived from immunohistochemistry was performed with the Kruskal-Wallis test and Dunn's multiple comparisons test, using the software system GraphPad Prism Version 5.0 (GraphPad Software, San Diego, CA, USA). The significance level was 0.05.

Statistical analysis of changes in gene expression was preceded by normalization using Affymetrix-Power-Tools and log2 transformation. Symmetry of the data was verified by mean versus average analysis and boxplots. As a first approach towards statistical data analysis, hierarchical clustering was performed using Spearman correlation coefficient as distance measurement. The ward-linkage method was used to generate dendrograms. Since technical replicates were clustered together in almost all cases, these replicates were averaged and the average was used in further condition clustering, separating samples from normal tissue and those that originated from tumors. Thereafter, separate data across all four timepoints was clustered per individual patient, allowing patients to be grouped into distinct partitions.

Separating normal colon tissue from CRC tissue samples, only patients from the largest partition group were used to compare intensity levels between presurgery and 10' postsurgery timepoints and presurgery and 45' postsurgery timepoints. T-tests were performed and p-values were calculated. Probe sets with p>0.001 were excluded as were probe sets with <2 log-fold changes in intensity. The overlap from both comparisons was identified as a list of genes that were particularly vulnerable to warm and cold ischemia. A list of all genes sorted according to the smallest coefficient of variation (CV) was used to identify genes that were apparently not vulnerable to warm and cold ischemia.

For detailed comparison between the three different cold ischemia time intervals, all samples taken at biopsy were excluded from the colorectal data pool. After exclusion, cluster dendrograms across the remaining three timepoints were generated, allowing patients to be grouped into distinct partitions. Patients from the largest partition group were used to compare intensity levels between presurgery and 10' postsurgery timepoint and presurgery and 45' postsurgery timepoint. T-tests were performed and p-values were calculated.

## SUPPLEMENTARY MATERIAL, FIGURES AND TABLES


