# Supplementary material for: Surgical procedures and postsurgical tissue processing significantly affect expression of genes and EGFR-pathway proteins in colorectal cancer tissue
Source: Oncotarget. 2014 Nov 3;5(22):11017–28. doi: 10.18632/oncotarget.2669 (PMC4294341; doi:10.18632/oncotarget.2669)
Supplement: Supplementary file 1 [file oncotarget-05-11017-s001.pdf]

Surgical procedures and postsurgical tissue processing significantly affect expression of genes and EGFR-pathway proteins in colorectal cancer tissue

Supplementary Material

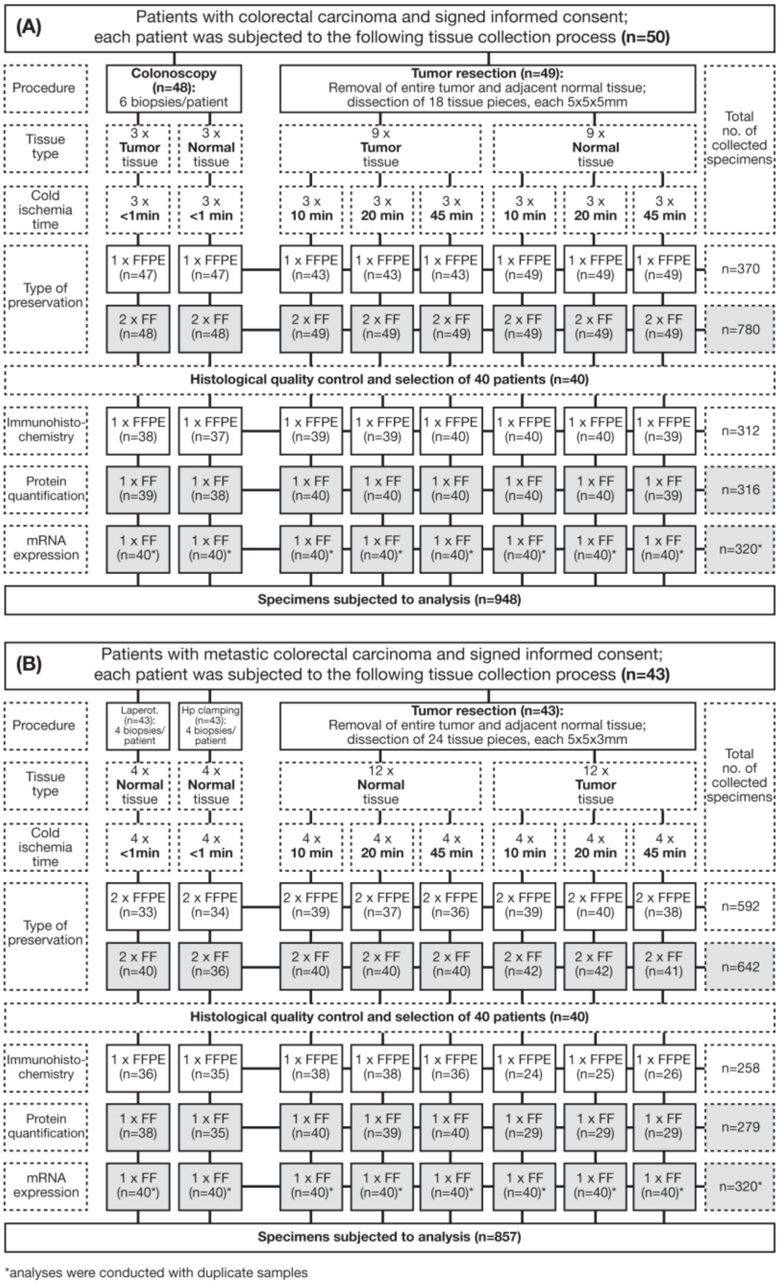

Supplementary Figure 1: Flow chart of patient enrollment and tissue collection. Patients had: (**A**) primary colorectal cancer; and (**B**) metastasized colorectal cancer. Some patients with metastasized cancer had hepatic pedicle clamping (H.p.). Tissue preservation methods were: frozen in liquid nitrogen (FF) or formalin-fixed paraffin-embedded (FFPE).

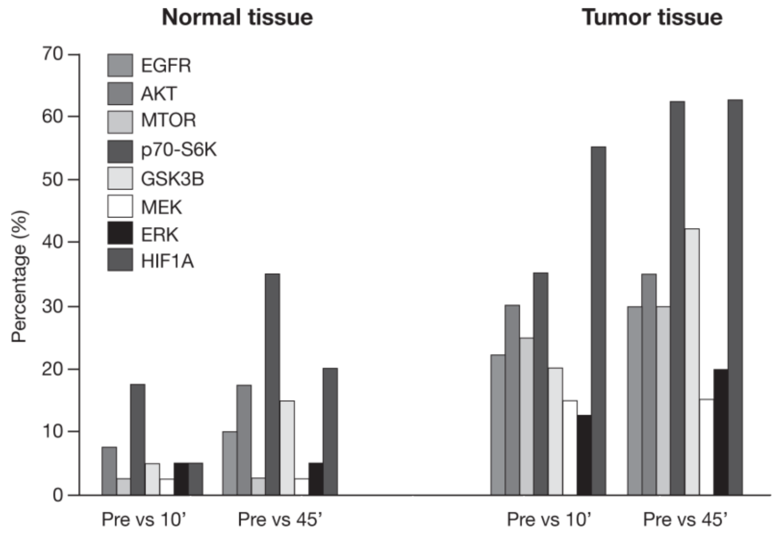

Supplementary Figure 2a: Protein expression in colon tissue. The figure shows changes of more than 2-fold in total protein expression of selected proteins, measured in normal colon and colorectal cancer tissue. Protein expression changes were compared: pre, before hepatic pedicle clamping; 10', 10 minutes after resection; and 45', 45 minutes after resection.

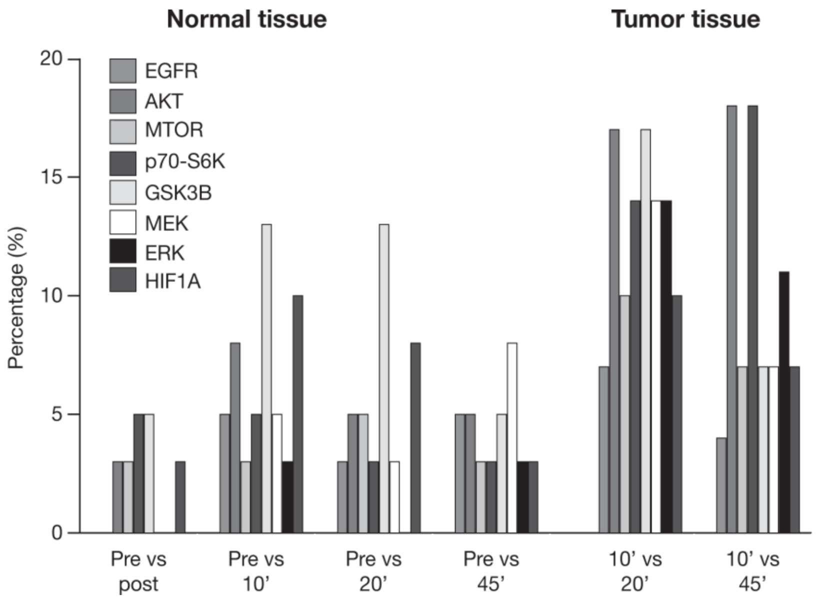

Supplementary Figure 2b: Protein expression in liver tissue. The figure shows changes of more than 2-fold in total protein expression of selected proteins, measured in normal hepatic and metastatic tissue. Protein expression changes were compared: pre, before hepatic pedicle clamping; post, after clamping; 10', 10 minutes after resection; 20', 20 minutes after resection; and 45', 45 minutes after resection.

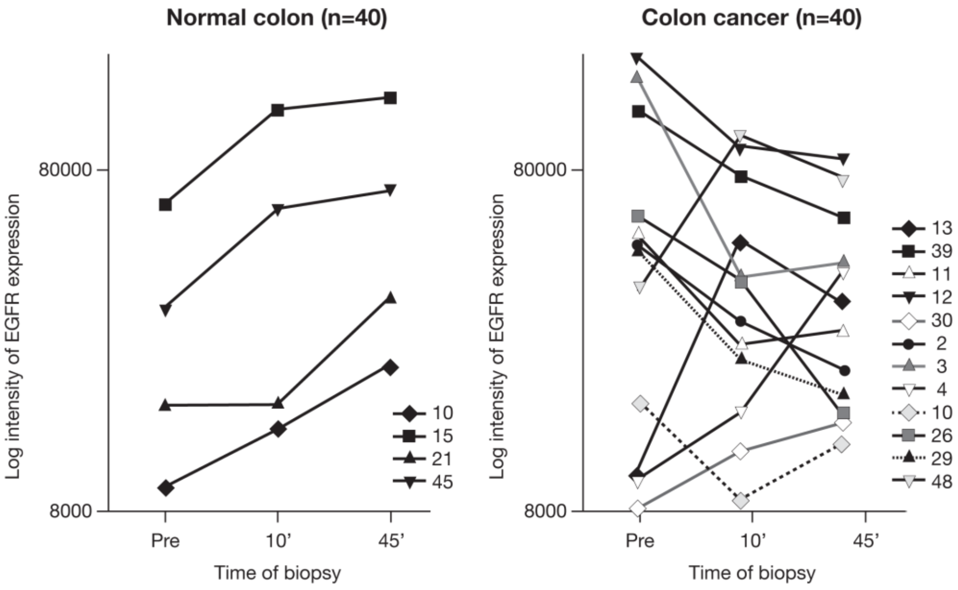

Supplementary Figure 3: Expression of EGFR in normal colon (left) and tumor tissue (right) in a subgroup of patients who showed at least 2-fold change in protein expression (up- or down-regulated) as determined by analyzing tissue lysates using MSD technology.

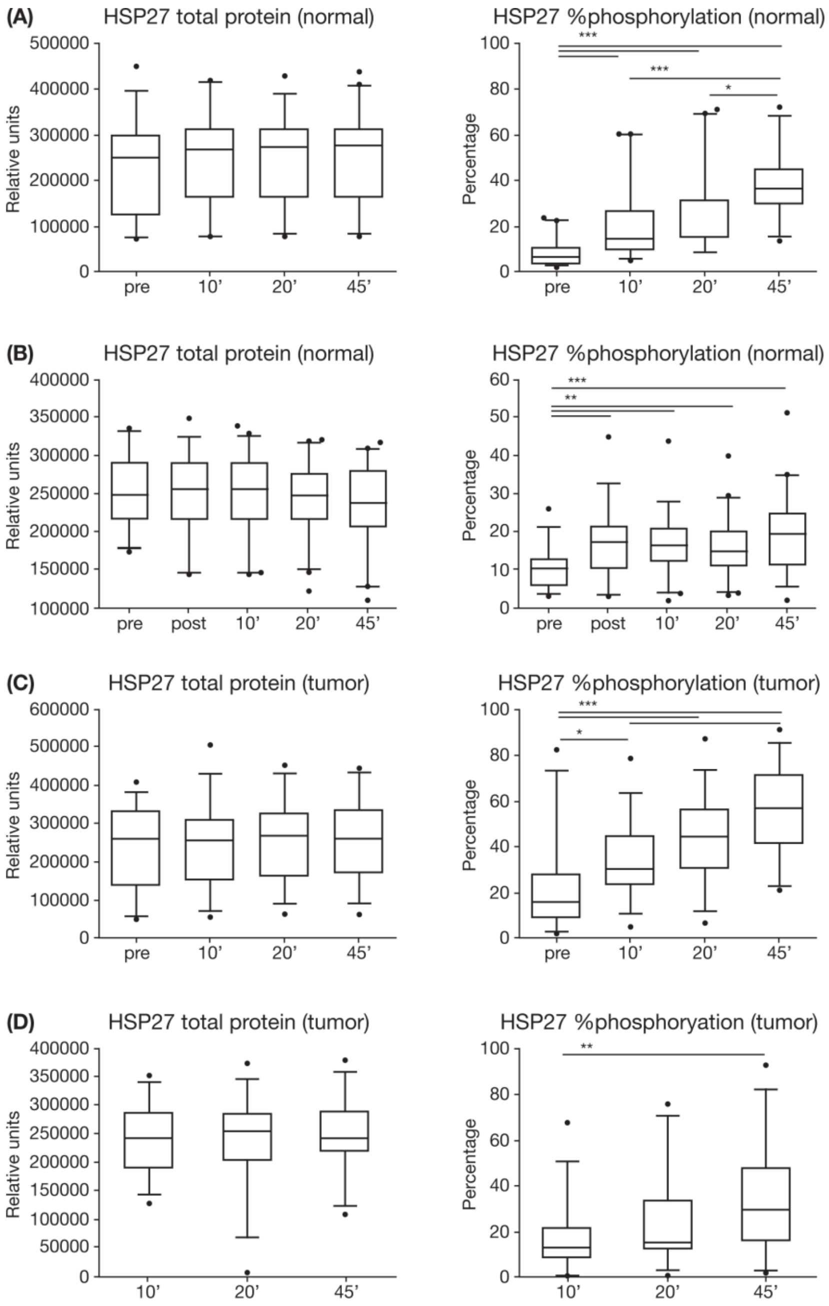

Supplementary Figure 4: Total protein expression (relative units) and percentage phosphorylation for HSP27 in: (**A**) normal colon; (**B**) liver; (**C**) primary colon cancer; and (**D**) metastatic liver lesion tissue. Tissue was obtained: pre, before hepatic pedicle clamping; post, after clamping; 10’, 10 minutes after resection; 20’, 20 minutes after resection; and 45’, 45 minutes after resection. *p<0.05; **p<0.01; ***p<0.001.

Supplementary Table 1: Differentially expressed genes in normal colon tissue according to when tissue was obtained. P-values represent the comparison between tissue obtained presurgery and 10 minutes after resection; the 70 regulated genes with ≥2-fold (statistically significant) change in expression are listed.

**Probe ID**	**Gene**	**Protein**	**p-value**	**Proportional change**
1555847_a_at	*LOC284454*	Hypothetical LOC284454	9.54667E-12	2.50
1555938_x_at	*VIM*	Vimentin	3.70146E-11	2.65
1557285_at	*AREGB*	Amphiregulin B	4.38611E-07	2.53
1564796_at	*EMP1*	Epithelial membrane protein 1	1.96927E-10	2.84
201041_s_at	*DUSP1*	Dual specificity phosphatase 1	1.20966E-17	3.60
201289_at	*CYR61*	Cysteine-rich, angiogenic inducer, 61	1.56579E-18	5.62
201631_s_at	*IER3*	Immediate early response 3	1.52502E-06	2.02
201693_s_at	*EGR1*	Early growth response 1	5.45124E-09	2.39
201694_s_at	*EGR1*	Early growth response 1	1.92369E-12	4.06
201739_at	*SGK1*	Serum/glucocorticoid regulated kinase 1	4.02631E-14	4.27
201893_x_at	*DCN*	Decorin	4.24098E-10	2.07
202291_s_at	*MGP*	Matrix GLA protein	3.32429E-08	3.16
202672_s_at	*ATF3*	Activating transcription factor 3	1.29365E-12	3.82
202988_s_at	*RGS1*	Regulator of G-protein signaling 1	4.20768E-21	5.07
203821_at	*HBEGF*	Heparin-binding EGF-like growth factor	1.92717E-10	3.35
204622_x_at	*NR4A2*	Nuclear receptor subfamily 4, group A, member 2	1.66002E-09	3.82
205200_at	*CLEC3B*	C-type lectin domain family 3, member B	1.65903E-11	2.38
205239_at	*AREG*	Amphiregulin	9.33122E-10	3.01
205382_s_at	*CFD*	Complement factor D (adipsin)	1.38175E-09	2.14
205440_s_at	*NPY1R*	Neuropeptide Y receptor Y1	6.18072E-10	2.17
206577_at	*VIP*	Vasoactive intestinal peptide	1.27434E-05	2.41
208078_s_at	*SIK1*	Salt-inducible kinase 1	8.60542E-09	3.15
208763_s_at	*TSC22D3*	TSC22 domain family, member 3	1.07833E-11	2.33
209101_at	*CTGF*	Connective tissue growth factor	7.37383E-16	3.06
209189_at	*FOS*	FBJ murine osteosarcoma viral oncogene homolog	1.26409E-18	6.81
209335_at	*DCN*	Decorin	1.20046E-10	2.34
210764_s_at	*CYR61*	Cysteine-rich, angiogenic inducer, 61	5.62682E-15	3.56
211896_s_at	*DCN*	Decorin	1.3672E-09	2.33
212225_at	*EIF1*	Eukaryotic translation initiation factor 1	3.33258E-14	2.06
212977_at	*CXCR7*	Chemokine (C-X-C motif) receptor 7	4.73779E-10	2.05
213068_at	*DPT*	Dermatopontin	7.0909E-10	2.52
214038_at	*CCL8*	Chemokine (C-C motif) ligand 8	5.52415E-14	2.84
216248_s_at	*NR4A2*	Nuclear receptor subfamily 4, group A, member 2	8.88079E-09	3.80
216834_at	*RGS1*	Regulator of G-protein signaling 1	1.65149E-22	5.04
218541_s_at	*C8orf4*	Chromosome 8 open reading frame 4	2.39257E-12	4.00
218730_s_at	*OGN*	Osteoglycin	1.11377E-08	2.36
219087_at	*ASPN*	Asporin	1.46805E-10	2.03

Probe ID	Gene	Protein	p-value	Proportional change
219230_at	*TMEM100*	Transmembrane protein 100	2.2327E-14	2.15
219295_s_at	*PCOLCE2*	Procollagen C-endopeptidase enhancer 2	3.16146E-08	2.13
220468_at	*ARL14*	ADP-ribosylation factor-like 14	7.67767E-11	2.10
222162_s_at	*ADAMTS1*	ADAM metallopeptidase with thrombospondin type 1 motif, 1	4.62579E-11	2.54
222722_at	*OGN*	Osteoglycin	1.34695E-08	2.79
223121_s_at	*SFRP2*	Secreted frizzled-related protein 2	1.04636E-08	2.18
223122_s_at	*SFRP2*	Secreted frizzled-related protein 2	1.07874E-09	3.21
224657_at	*ERRFI1*	ERBB receptor feedback inhibitor 1	7.42432E-10	2.09
227099_s_at	*C11orf96*	Chromosome 11 open reading frame 96	7.20862E-12	3.09
227404_s_at	*EGR1*	Early growth response 1	1.39748E-10	3.94
227697_at	*SOCS3*	Suppressor of cytokine signaling 3	0.000387697	2.15
228335_at	*CLDN11*	Claudin 11	1.03247E-10	2.32
230494_at	---	---	2.71373E-11	2.40
243509_at	---	---	5.3854E-16	2.13
36711_at	*MAFF*	V-maf musculoaponeurotic fibrosarcoma oncogene homolog F (avian)	1.6224E-09	2.66
38037_at	*HBEGF*	Heparin-binding EGF-like growth factor	2.60564E-09	2.50
1554436_a_at	*REG4*	Regenerating islet-derived family, member 4	0.000125463	-2.88
203649_s_at	*PLA2G2A*	Phospholipase A2, group IIA (platelets, synovial fluid)	5.92788E-06	-2.42
205844_at	*VNN1*	Vanin 1	0.000102012	-3.07
207214_at	*SPINK4*	Serine peptidase inhibitor, Kazal type 4	0.000340689	-2.75
207397_s_at	*HOXD13*	Homeobox D13	9.4269E-08	-2.95
212531_at	*LCN2*	Lipocalin 2	1.15271E-06	-2.68
212768_s_at	*OLFM4*	Olfactomedin 4	0.003466089	-2.27
214604_at	*HOXD11*	Homeobox D11	4.34958E-09	-2.12
219727_at	*DUOX2*	Dual oxidase 2	3.35786E-08	-3.69
219795_at	*SLC6A14*	Solute carrier family 6 (amino acid transporter), member 14	1.0087E-05	-3.66
221091_at	*INSL5*	Insulin-like 5	5.07532E-05	-2.69
223447_at	*REG4*	Regenerating islet-derived family, member 4	0.000132695	-2.50
228592_at	*MS4A1*	Membrane-spanning 4-domains, subfamily A, member 1	0.000474385	-2.21
229152_at	*C4orf7*	Chromosome 4 open reading frame 7	0.001397357	-2.49
229400_at	*HOXD10*	Homeobox D10	8.95393E-08	-2.89
236681_at	*HOXD13*	Homeobox D13	5.02826E-08	-2.05
238847_at	---	---	4.27233E-08	-2.94

Supplementary Table 2: Differentially expressed genes in normal colon tissue according to a comparison of timepoint. P-values represent the comparison between tissue obtained presurgery and 10 minutes after resection; the 96 regulated genes with ≥2-fold (statistically significant) change in expression are listed.

**Probe ID**	**Gene**	**Protein**	**p-value**	**Proportional change**
1552296_at	*BEST4*	Bestrophin 4	1.28199E-06	2.09
1555847_a_at	*LOC284454*	Hypothetical LOC284454	1.49773E-14	2.17
1555938_x_at	*VIM*	Vimentin	1.98675E-18	2.53
1557285_at	*AREGB*	Amphiregulin B	6.55772E-09	2.35
1564796_at	*EMP1*	Epithelial membrane protein 1	7.68089E-17	3.82
201041_s_at	*DUSP1*	Dual specificity phosphatase 1	2.99931E-23	4.45
201289_at	*CYR61*	Cysteine-rich, angiogenic inducer, 61	2.83776E-22	5.86
201324_at	*EMP1*	Epithelial membrane protein 1	9.86918E-12	2.09
201325_s_at	*EMP1*	Epithelial membrane protein 1	1.90042E-11	2.09
201465_s_at	*JUN*	Jun proto-oncogene	6.16667E-17	2.02
201466_s_at	*JUN*	Jun proto-oncogene	4.02899E-21	2.43
201631_s_at	*IER3*	Immediate early response 3	1.04871E-08	2.03
201693_s_at	*EGR1*	Early growth response 1	1.42355E-12	2.60
201694_s_at	*EGR1*	Early growth response 1	1.79272E-18	4.78
201739_at	*SGK1*	Serum/glucocorticoid regulated kinase 1	2.74262E-16	4.62
201893_x_at	*DCN*	Decorin	2.54325E-11	2.21
202068_s_at	*LDLR*	Low density lipoprotein receptor	1.13748E-08	2.06
202241_at	*TRIB1*	Tribbles homolog 1 (Drosophila)	1.49548E-14	2.00
202291_s_at	*MGP*	Matrix GLA protein	4.90017E-09	3.44
202672_s_at	*ATF3*	Activating transcription factor 3	7.34522E-15	3.76
202988_s_at	*RGS1*	Regulator of G-protein signaling 1	5.04504E-25	5.24
202995_s_at	*FBLN1*	Fibulin 1	6.17794E-08	2.02
203821_at	*HBEGF*	Heparin-binding EGF-like growth factor	4.06679E-13	3.31
203851_at	*IGFBP6*	Insulin-like growth factor binding protein 6	2.56813E-12	2.00
204622_x_at	*NR4A2*	Nuclear receptor subfamily 4, group A, member 2	1.23506E-10	3.38
204955_at	*SRPX*	Sushi-repeat-containing protein, X-linked	4.15751E-09	2.10
205200_at	*CLEC3B*	C-type lectin domain family 3, member B	2.07874E-12	2.49
205239_at	*AREG*	Amphiregulin	4.65456E-10	2.78
205382_s_at	*CFD*	Complement factor D (adipsin)	4.48062E-10	2.18
205440_s_at	*NPY1R*	Neuropeptide Y receptor Y1	7.56685E-12	2.41
205799_s_at	*SLC3A1*	Solute carrier family 3, member 1	2.1179E-07	2.25
206577_at	*VIP*	Vasoactive intestinal peptide	7.96108E-06	2.47
207977_s_at	*DPT*	Dermatopontin	7.55307E-12	2.04
208078_s_at	*SIK1*	Salt-inducible kinase 1	2.81573E-09	2.88
208763_s_at	*TSC22D3*	TSC22 domain family, member 3	1.50914E-14	2.33
209101_at	*CTGF*	Connective tissue growth factor	2.93886E-17	3.21
209189_at	*FOS*	FBJ murine osteosarcoma viral oncogene	9.30065E-25	9.15

Probe ID	Gene	Protein	p-value	Proportional change
		homolog		
209335_at	*DCN*	Decorin	8.07853E-12	2.45
210472_at	*MT1G*	Metallothionein 1G	1.31269E-08	2.25
210764_s_at	*CYR61*	Cysteine-rich, angiogenic inducer, 61	1.7157E-19	3.80
211813_x_at	*DCN*	Decorin	3.33982E-11	2.13
211896_s_at	*DCN*	Decorin	5.66051E-11	2.53
211998_at	*H3F3B*	H3 histone, family 3B (H3.3B)	2.99953E-23	2.20
212099_at	*RHOB*	Ras homolog gene family, member B	9.0115E-14	2.19
212225_at	*EIF1*	Eukaryotic translation initiation factor 1	3.70056E-19	2.60
212977_at	*CXCR7*	Chemokine (C-X-C motif) receptor 7	3.00534E-13	2.13
213068_at	*DPT*	Dermatopontin	1.96475E-11	2.81
213071_at	*DPT*	Dermatopontin	1.93102E-10	2.04
214038_at	*CCL8*	Chemokine (C-C motif) ligand 8	4.23919E-15	3.11
216248_s_at	*NR4A2*	Nuclear receptor subfamily 4, group A, member 2	2.09133E-10	3.49
216834_at	*RGS1*	Regulator of G-protein signaling 1	8.9595E-25	5.14
218541_s_at	*C8orf4*	Chromosome 8 open reading frame 4	3.6358E-15	4.03
218730_s_at	*OGN*	Osteoglycin	5.34811E-09	2.40
219087_at	*ASPN*	Asporin	5.37941E-11	2.08
219230_at	*TMEM100*	Transmembrane protein 100	6.16856E-15	2.26
219295_s_at	*PCOLCE2*	Procollagen C-endopeptidase enhancer 2	8.72205E-10	2.41
220037_s_at	*LYVE1*	Lymphatic vessel endothelial hyaluronan receptor 1	1.23282E-11	2.07
220468_at	*ARL14*	ADP-ribosylation factor-like 14	3.09932E-13	2.18
222162_s_at	*ADAMTS1*	ADAM metallopeptidase with thrombospondin type 1 motif, 1	7.41382E-12	2.51
222722_at	*OGN*	Osteoglycin	7.9114E-09	2.82
222943_at	*GBA3*	Glucosidase, beta, acid 3 (cytosolic)	6.3056E-07	2.33
223121_s_at	*SFRP2*	Secreted frizzled-related protein 2	9.25419E-09	2.18
223122_s_at	*SFRP2*	Secreted frizzled-related protein 2	5.51482E-10	3.32
224657_at	*ERRFI1*	ERBB receptor feedback inhibitor 1	1.2124E-12	2.12
225664_at	*COL12A1*	Collagen, type XII, alpha 1	7.50679E-10	2.03
225767_at	*LOC284801*	Hypothetical protein LOC284801	2.83155E-14	2.18
227099_s_at	*C11orf96*	Chromosome 11 open reading frame 96	1.42997E-16	3.18
227404_s_at	*EGR1*	Early growth response 1	2.56676E-16	5.01
227697_at	*SOCS3*	Suppressor of cytokine signaling 3	1.39534E-06	2.28
227827_at	---	---	0.000197723	2.05
228335_at	*CLDN11*	Claudin 11	5.1725E-11	2.41
228885_at	*MAMDC2*	MAM domain containing 2	4.88713E-09	2.07
230494_at	---	---	8.58621E-15	2.65
243509_at	---	---	4.91602E-16	2.40
36711_at	*MAFF*	V-maf musculoaponeurotic fibrosarcoma oncogene homolog F (avian)	1.1619E-10	2.57
38037_at	*HBEGF*	Heparin-binding EGF-like growth factor	3.03719E-12	2.46
1554436_a_at	*REG4*	Regenerating islet-derived family, member 4	3.97492E-05	-3.18

Probe ID	Gene	Protein	p-value	Proportional change
1558549_s_at	*VNN1*	Vanin 1	1.46257E-06	-2.10
203649_s_at	*PLA2G2A*	Phospholipase A2, group IIA (platelets, synovial fluid)	1.56184E-07	-2.94
205242_at	*CXCL13*	Chemokine (C-X-C motif) ligand 13	0.001696161	-2.09
205844_at	*VNN1*	Vanin 1	1.64394E-07	-4.50
207214_at	*SPINK4*	Serine peptidase inhibitor, Kazal type 4	0.00027595	-2.87
207397_s_at	*HOXD13*	Homeobox D13	5.32093E-11	-3.44
212531_at	*LCN2*	Lipocalin 2	7.01758E-09	-3.24
212768_s_at	*OLFM4*	Olfactomedin 4	0.000639115	-2.79
214604_at	*HOXD11*	Homeobox D11	1.29845E-11	-2.32
219727_at	*DUOX2*	Dual oxidase 2	2.23839E-10	-4.40
219795_at	*SLC6A14*	Solute carrier family 6 (amino acid transporter), member 14	3.60189E-07	-4.45
221091_at	*INSL5*	Insulin-like 5	3.79746E-07	-3.25
223447_at	*REG4*	Regenerating islet-derived family, member 4	2.84257E-05	-2.78
228592_at	*MS4A1*	Membrane-spanning 4-domains, subfamily A, member 1	9.1242E-05	-2.50
229152_at	*C4orf7*	Chromosome 4 open reading frame 7	0.000120605	-3.02
229400_at	*HOXD10*	Homeobox D10	4.46316E-10	-3.26
236681_at	*HOXD13*	Homeobox D13	2.53117E-11	-2.30
238847_at	---	---	7.09608E-11	-3.50
238999_at	---	---	7.58656E-17	-2.04

Supplementary Table 3: Differentially expressed genes in colon tumor tissue according to a comparison of timepoint. P-values represent the comparison between tissue obtained presurgery and 10 minutes after resection; the 178 regulated genes with ≥2-fold (statistically significant) change in expression are listed.

Probe ID	Gene	Protein	p-value	Proportional change
1555724_s_at	*TAGLN*	Transgelin	4.73342E-05	2.14
1555938_x_at	*VIM*	Vimentin	6.5247E-06	2.06
1568574_x_at	*SPP1*	Secreted phosphoprotein 1	0.002506207	2.02
201058_s_at	*MYL9*	Myosin, light chain 9, regulatory	9.1293E-05	2.15
201147_s_at	*TIMP3*	TIMP metallopeptidase inhibitor 3	1.4351E-05	2.15
201150_s_at	*TIMP3*	TIMP metallopeptidase inhibitor 3	1.77334E-05	2.03
201289_at	*CYR61*	Cysteine-rich, angiogenic inducer, 61	1.33434E-06	2.67
201496_x_at	*MYH11*	Myosin, heavy chain 11, smooth muscle	0.000639767	2.02
201645_at	*TNC*	Tenascin C	0.000236339	2.29
201792_at	*AEBP1*	AE binding protein 1	6.8465E-05	2.03
202237_at	*NNMT*	Nicotinamide N-methyltransferase	2.57535E-05	2.15
202274_at	*ACTG2*	Actin, gamma 2, smooth muscle, enteric	7.506E-05	2.03
202291_s_at	*MGP*	Matrix GLA protein	1.93448E-05	2.77
202310_s_at	*COL1A1*	Collagen, type I, alpha 1	0.001917905	2.05
202311_s_at	*COL1A1*	Collagen, type I, alpha 1	0.002116429	2.09
202363_at	*SPOCK1*	Sparc/osteonectin, cwcv and kazal-like domains proteoglycan (testican) 1	3.37525E-05	2.19
202437_s_at	*CYP1B1*	Cytochrome P450, family 1, subfamily B, polypeptide 1	0.001662327	2.11
202498_s_at	*SLC2A3*	Solute carrier family 2 (facilitated glucose transporter), member 3	0.000122207	2.00
202499_s_at	*SLC2A3*	Solute carrier family 2 (facilitated glucose transporter), member 3	0.000238055	2.19
202672_s_at	*ATF3*	Activating transcription factor 3	2.47058E-05	2.02
202766_s_at	*FBN1*	Fibrillin 1	5.74137E-05	2.08
202988_s_at	*RGS1*	Regulator of G-protein signaling 1	1.81321E-09	2.97
203083_at	*THBS2*	Thrombospondin 2	5.10405E-05	2.70
204051_s_at	*SFRP4*	Secreted frizzled-related protein 4	1.8344E-07	3.83
204052_s_at	*SFRP4*	Secreted frizzled-related protein 4	2.86372E-07	3.32
204457_s_at	*GAS1*	Growth arrest-specific 1	0.000455298	2.82
204472_at	*GEM*	GTP binding protein overexpressed in skeletal muscle	4.61815E-08	2.50
204622_x_at	*NR4A2*	Nuclear receptor subfamily 4, group A, member 2	2.35455E-05	2.13
204939_s_at	*PLN*	Phospholamban	5.48691E-05	2.10
204940_at	*PLN*	Phospholamban	5.82032E-05	2.11
205422_s_at	*ITGBL1*	Integrin, beta-like 1 (with EGF-like repeat domains)	1.29825E-05	2.91
205547_s_at	*TAGLN*	Transgelin	0.000109081	2.26
205713_s_at	*COMP*	Cartilage oligomeric matrix protein	1.01742E-05	2.48
205941_s_at	*COL10A1*	Collagen, type X, alpha 1	9.47934E-05	2.38
206224_at	*CST1*	Cystatin SN	0.004959398	2.06
206577_at	*VIP*	Vasoactive intestinal peptide	0.002985188	2.10

Probe ID	Gene	Protein	p-value	Proportional change
207173_x_at	*CDH11*	Cadherin 11, type 2, OB-cadherin (osteoblast)	8.60461E-05	2.06
209875_s_at	*SPP1*	Secreted phosphoprotein 1	0.001536153	2.74
210004_at	*OLR1*	Oxidized low density lipoprotein (lectin-like) receptor 1	0.000130964	2.09
210511_s_at	*INHBA*	Inhibin, beta A	2.07614E-05	2.44
210517_s_at	*AKAP12*	A kinase (PRKA) anchor protein 12	1.56563E-05	2.04
210764_s_at	*CYR61*	Cysteine-rich, angiogenic inducer, 61	2.62979E-06	2.40
211122_s_at	*CXCL11*	Chemokine (C-X-C motif) ligand 11	0.006808687	2.10
212344_at	*SULF1*	Sulfatase 1	2.89487E-05	2.21
212353_at	*SULF1*	Sulfatase 1	9.09842E-06	2.64
212354_at	*SULF1*	Sulfatase 1	1.30312E-05	2.41
213905_x_at	*BGN*	Biglycan	0.000330535	2.05
213943_at	*TWIST1*	Twist homolog 1 (Drosophila)	8.58632E-05	2.25
215033_at	*TM4SF1*	Transmembrane 4 L six family member 1	1.2279E-08	2.04
215646_s_at	*VCAN*	Versican	0.000677785	2.17
216005_at	*TNC*	Tenascin C	4.03919E-05	2.39
216248_s_at	*NR4A2*	Nuclear receptor subfamily 4, group A, member 2	1.26792E-05	2.29
216834_at	*RGS1*	Regulator of G-protein signaling 1	3.05537E-09	2.68
217428_s_at	*COL10A1*	Collagen, type X, alpha 1	0.000123852	3.64
218468_s_at	*GREM1*	Gremlin 1	0.000227583	2.72
218469_at	*GREM1*	Gremlin 1	0.0003666	2.59
219087_at	*ASPN*	Asporin	2.23181E-05	2.92
221730_at	*COL5A2*	Collagen, type V, alpha 2	0.000692635	2.01
221748_s_at	*TNS1*	Tensin 1	0.000133411	2.03
222877_at	---	---	1.4318E-06	2.14
223121_s_at	*SFRP2*	Secreted frizzled-related protein 2	0.000178028	2.59
223122_s_at	*SFRP2*	Secreted frizzled-related protein 2	9.3884E-05	3.20
223475_at	*CRISPLD1*	Cysteine-rich secretory protein LCCL domain containing 1	1.07185E-05	2.17
224396_s_at	*ASPN*	Asporin	6.15766E-06	2.54
224694_at	*ANTXR1*	Anthrax toxin receptor 1	0.000102969	2.25
225381_at	*LOC399959*	Hypothetical LOC399959	4.40169E-05	2.44
225481_at	*FRMD6*	FERM domain containing 6	2.27148E-05	2.02
225664_at	*COL12A1*	Collagen, type XII, alpha 1	0.000310454	2.18
225681_at	*CTHRC1*	Collagen triple helix repeat containing 1	7.13833E-05	2.79
225762_x_at	*LOC284801*	Hypothetical protein LOC284801	1.00682E-12	2.54
225767_at	*LOC284801*	Hypothetical protein LOC284801	7.6829E-15	4.02
225782_at	*MSRB3*	Methionine sulfoxide reductase B3	0.00035065	2.01
226237_at	*COL8A1*	Collagen, type VIII, alpha 1	9.79999E-06	3.10
226777_at	*ADAM12*	ADAM metallopeptidase domain 12	0.00044622	2.28
226930_at	*FNDC1*	Fibronectin type III domain containing 1	2.64629E-06	3.01
227099_s_at	*C11orf96*	Chromosome 11 open reading frame 96	1.68701E-06	2.48
227140_at	*INHBA*	Inhibin, beta A	4.01738E-05	2.97

Probe ID	Gene	Protein	p-value	Proportional change
227235_at	*GUCY1A3*	Guanylate cyclase 1, soluble, alpha 3	7.34799E-05	2.11
227399_at	*VGLL3*	Vestigial like 3 (Drosophila)	0.000940894	2.06
227566_at	*NTM*	Neurotrimin	8.58912E-05	2.26
227697_at	*SOCS3*	Suppressor of cytokine signaling 3	0.000574813	2.07
228202_at	*PLN*	Phospholamban	3.4219E-05	2.35
228640_at	*PCDH7*	Protocadherin 7	0.000418283	2.04
229271_x_at	*COL11A1*	Collagen, type XI, alpha 1	0.000948907	2.06
229554_at	---	---	0.000145241	2.17
229802_at	---	---	0.000922503	2.00
230493_at	*SHISA2*	Shisa homolog 2 (*Xenopus laevis*)	0.000179494	2.01
231766_s_at	*COL12A1*	Collagen, type XII, alpha 1	0.000379392	2.28
231879_at	*COL12A1*	Collagen, type XII, alpha 1	0.000216652	2.29
232113_at	---	---	3.02703E-05	2.39
235944_at	*HMCN1*	Hemicentin 1	7.63204E-06	2.17
236179_at	*CDH11*	Cadherin 11, type 2, OB-cadherin (osteoblast)	8.87507E-05	2.35
238481_at	*MGP*	Matrix GLA protein	1.18316E-06	2.53
238623_at	---	---	1.04654E-06	2.44
37892_at	*COL11A1*	Collagen, type XI, alpha 1	0.000101146	2.79
1553828_at	*FAM55A*	Family with sequence similarity 55, member A	0.000633162	-2.06
1554436_a_at	*REG4*	Regenerating islet-derived family, member 4	0.028007727	-2.52
1555962_at	*B3GNT7*	UDP-GlcNAc:betaGal beta-1,3-N-acetylglucosaminyltransferase 7	0.000795644	-2.54
1555963_x_at	*B3GNT7*	UDP-GlcNAc:betaGal beta-1,3-N-acetylglucosaminyltransferase 7	0.000789723	-2.87
203240_at	*FCGBP*	Fc fragment of IgG binding protein	0.002948685	-2.90
203649_s_at	*PLA2G2A*	Phospholipase A2, group IIA (platelets, synovial fluid)	0.003760504	-2.42
203691_at	*PI3*	Peptidase inhibitor 3, skin-derived	0.002462861	-2.10
203908_at	*SLC4A4*	Solute carrier family 4, sodium bicarbonate cotransporter, member 4	0.000983189	-3.03
204018_x_at	*HBA1 /// HBA2*	Hemoglobin, alpha 1 /// hemoglobin, alpha 2	0.000119014	-2.03
204213_at	*PIGR*	Polymeric immunoglobulin receptor	0.000398215	-2.70
204673_at	*MUC2*	Mucin 2, oligomeric mucus/gel-forming	0.005678468	-2.64
204818_at	*HSD17B2*	Hydroxysteroid (17-beta) dehydrogenase 2	0.000119211	-2.26
205097_at	*SLC26A2*	Solute carrier family 26 (sulfate transporter), member 2	0.002478216	-2.39
205185_at	*SPINK5*	Serine peptidase inhibitor, Kazal type 5	5.58358E-05	-2.29
205892_s_at	*FABP1*	Fatty acid binding protein 1, liver	0.032075593	-2.35
205950_s_at	*CA1*	Carbonic anhydrase I	2.96487E-05	-3.66
205979_at	*SCGB2A1*	Secretoglobin, family 2A, member 1	0.000787599	-2.38
206000_at	*MEP1A*	Meprin A, alpha (PABA peptide hydrolase)	0.006828159	-2.10
206143_at	*SLC26A3*	Solute carrier family 26, member 3	0.002087168	-4.31
206198_s_at	*CEACAM7*	Carcinoembryonic antigen-related cell adhesion molecule 7	0.000743488	-3.77
206199_at	*CEACAM7*	Carcinoembryonic antigen-related cell adhesion molecule 7	0.000617427	-3.11
206208_at	*CA4*	Carbonic anhydrase IV	0.000142793	-2.22

Probe ID	Gene	Protein	p-value	Proportional change
206209_s_at	*CA4*	Carbonic anhydrase IV	5.04159E-05	-2.98
206262_at	*ADH1C*	Alcohol dehydrogenase 1C (class I), gamma polypeptide	5.40013E-05	-3.66
206268_at	*LEFTY1*	Left-right determination factor 1	0.018432403	-2.25
206561_s_at	*AKR1B10*	Aldo-keto reductase family 1, member B10 (aldose reductase)	0.000169098	-2.19
206641_at	*TNFRSF17*	Tumor necrosis factor receptor superfamily, member 17	7.48607E-05	-2.02
206664_at	*SI*	Sucrase-isomaltase (alpha-glucosidase)	9.10874E-05	-5.30
207003_at	*GUCA2A*	Guanylate cyclase activator 2A (guanylin)	2.67886E-05	-2.88
207214_at	*SPINK4*	Serine peptidase inhibitor, Kazal type 4	0.006152697	-2.96
207245_at	*UGT2B17*	UDP glucuronosyltransferase 2 family, polypeptide B17	0.002178709	-3.43
208450_at	*LGALS2*	Lectin, galactoside-binding, soluble, 2	1.96728E-05	-2.58
209116_x_at	*HBB*	Hemoglobin, beta	2.82831E-05	-2.46
209301_at	*CA2*	Carbonic anhydrase II	0.000491151	-2.82
209374_s_at	*IGHM*	Immunoglobulin heavy constant mu	0.002618513	-2.23
209458_x_at	*HBA1 /// HBA2*	Hemoglobin, alpha 1 /// hemoglobin, alpha 2	9.34895E-05	-2.12
209613_s_at	*ADH1B*	Alcohol dehydrogenase 1B (class I), beta polypeptide	0.006916787	-2.22
210107_at	*CLCA1*	Chloride channel accessory 1	0.000416754	-4.22
210738_s_at	*SLC4A4*	Solute carrier family 4, sodium bicarbonate cotransporter, member 4	0.000383647	-2.11
211696_x_at	*HBB*	Hemoglobin, beta	1.18856E-05	-2.30
211699_x_at	*HBA1 /// HBA2*	Hemoglobin, alpha 1 /// hemoglobin, alpha 2	0.000128052	-2.02
211745_x_at	*HBA1 /// HBA2*	Hemoglobin, alpha 1 /// hemoglobin, alpha 2	8.1156E-05	-2.16
211848_s_at	*CEACAM7*	Carcinoembryonic antigen-related cell adhesion molecule 7	0.000588658	-3.12
212531_at	*LCN2*	Lipocalin 2	0.002337585	-2.20
212592_at	*IGJ*	Immunoglobulin J polypeptide, linker protein for immunoglobulin alpha and mu polypeptides	0.000180401	-3.69
212768_s_at	*OLFM4*	Olfactomedin 4	0.001184695	-4.23
214142_at	*ZG16*	Zymogen granule protein 16 homolog (rat)	0.000149205	-4.79
214414_x_at	*HBA1 /// HBA2*	Hemoglobin, alpha 1 /// hemoglobin, alpha 2	2.72256E-05	-2.47
214433_s_at	*SELENBP1*	Selenium binding protein 1	0.000985865	-2.04
214598_at	*CLDN8*	Claudin 8	1.78118E-05	-4.17
215657_at	*SLC26A3*	Solute carrier family 26, member 3	0.008000304	-2.03
217022_s_at	*IGHA1 /// IGHA2*	Immunoglobulin heavy constant alpha 1 /// immunoglobulin heavy	0.000214466	-3.22
	LOC100126583	/// hypothetical LOC100126583		
217109_at	*MUC4*	Mucin 4, cell surface associated	0.000620192	-2.84
217110_s_at	*MUC4*	Mucin 4, cell surface associated	0.000989039	-2.48
217232_x_at	*HBB*	Hemoglobin, beta	2.09662E-05	-2.04
217414_x_at	*HBA1 /// HBA2*	Hemoglobin, alpha 1 /// hemoglobin, alpha 2	5.94249E-05	-2.17
217546_at	*MT1M*	Metallothionein 1M	0.002091263	-2.47
219727_at	*DUOX2*	Dual oxidase 2	0.000497377	-2.84
219795_at	*SLC6A14*	Solute carrier family 6 (amino acid transporter), member 14	0.010883367	-2.29
219948_x_at	*UGT2A3*	UDP glucuronosyltransferase 2 family, polypeptide A3	0.001187812	-3.07
220026_at	*CLCA4*	Chloride channel accessory 4	4.4668E-05	-5.04

Probe ID	Gene	Protein	p-value	Proportional change
220376_at	*LRRC19*	Leucine rich repeat containing 19	0.001283828	-2.46
220834_at	*MS4A12*	Membrane-spanning 4-domains, subfamily A, member 12	1.03895E-05	-4.65
223447_at	*REG4*	Regenerating islet-derived family, member 4	0.027839534	-2.23
223597_at	*ITLN1*	Intelectin 1 (galactofuranose binding)	0.000148222	-4.89
224027_at	*CCL28*	Chemokine (C-C motif) ligand 28	4.55114E-05	-2.27
224412_s_at	*TRPM6*	Transient receptor potential cation channel, subfamily M, member 6	0.000748472	-2.26
224959_at	*SLC26A2*	Solute carrier family 26 (sulfate transporter), member 2	0.004868048	-2.10
224963_at	*SLC26A2*	Solute carrier family 26 (sulfate transporter), member 2	0.002598016	-2.08
226147_s_at	*PIGR*	Polymeric immunoglobulin receptor	0.01084721	-2.54
226654_at	*MUC12*	Mucin 12, cell surface associated	0.009193088	-2.03
227725_at	*ST6GALNAC1*	ST6 (alpha-N-acetyl-neuraminyl-2,3-beta-galactosyl-1,3	0.004737468	-2.06
227735_s_at	*C10orf99*	Chromosome 10 open reading frame 99	0.004911848	-2.04
227736_at	*C10orf99*	Chromosome 10 open reading frame 99	0.00861509	-2.22
228232_s_at	*VSIG2*	V-set and immunoglobulin domain containing 2	0.000105191	-2.57
228241_at	*AGR3*	Anterior gradient homolog 3 (*Xenopus laevis*)	0.023109441	-2.16
229070_at	*C6orf105*	Chromosome 6 open reading frame 105	9.90567E-06	-3.45
229254_at	*MFSD4*	Major facilitator superfamily domain containing 4	0.003950209	-2.16
234632_x_at	---	---	0.000302489	-2.79
238143_at	*LOC646627*	Phospholipase inhibitor	0.001739348	-2.10
238750_at	*CCL28*	Chemokine (C-C motif) ligand 28	7.86416E-05	-2.43
239673_at	---	---	0.000459064	-2.00
242601_at	*HEPACAM2*	HEPACAM family member 2	0.001268202	-3.43

Supplementary Table 4: Differentially expressed genes in colon tumor tissue according to a comparison of timepoint. P-values represent the comparison between tissue obtained presurgery and 45 minutes after resection; the 332 regulated genes with ≥2-fold (statistically significant) change in expression are listed.

**Probe ID**	**Gene**	**Protein**	**P-value**	**Proportional change**
1552309_a_at	*NEXN*	Nexilin (F actin binding protein)	8.64128E-07	2.21
1554018_at	*GPNMB*	Glycoprotein (transmembrane) nmb	5.28765E-06	2.18
1554741_s_at	*FGF7 /// KGFLP1*	Fibroblast growth factor 7 /// keratinocyte growth factor-protein 1	0.000151345	2.18
	/// KGFLP2	/// keratinocyte growth factor-like protein 2		
1555229_a_at	*C1S*	Complement component 1, s subcomponent	4.3929E-06	2.26
1555724_s_at	*TAGLN*	Transgelin	6.24171E-07	2.86
1555778_a_at	*POSTN*	Periostin, osteoblast specific factor	0.002106898	2.38
1556325_at	*FILIP1*	Filamin A interacting protein 1	8.51026E-06	2.11
1568574_x_at	*SPP1*	Secreted phosphoprotein 1	1.022E-05	2.66
200986_at	*SERPING1*	Serpin peptidase inhibitor, clade G (C1 inhibitor), member 1	4.66163E-08	2.39
201041_s_at	*DUSP1*	Dual specificity phosphatase 1	1.14772E-05	2.21
201058_s_at	*MYL9*	Myosin, light chain 9, regulatory	2.00947E-06	2.92
201109_s_at	*THBS1*	Thrombospondin 1	3.33848E-06	2.16
201110_s_at	*THBS1*	Thrombospondin 1	5.94571E-06	2.19
201141_at	*GPNMB*	Glycoprotein (transmembrane) nmb	1.30308E-06	2.30
201147_s_at	*TIMP3*	TIMP metallopeptidase inhibitor 3	5.34657E-08	2.47
201150_s_at	*TIMP3*	TIMP metallopeptidase inhibitor 3	3.1703E-08	2.37
201261_x_at	*BGN*	Biglycan	4.85485E-06	2.12
201289_at	*CYR61*	Cysteine-rich, angiogenic inducer, 61	3.96116E-08	3.01
201430_s_at	*DPYSL3*	Dihydropyrimidinase-like 3	4.8262E-09	2.46
201431_s_at	*DPYSL3*	Dihydropyrimidinase-like 3	1.33119E-08	2.41
201496_x_at	*MYH11*	Myosin, heavy chain 11, smooth muscle	4.17357E-06	3.59
201497_x_at	*MYH11*	Myosin, heavy chain 11, smooth muscle	6.93199E-06	2.78
201616_s_at	*CALD1*	Caldesmon 1	6.51618E-06	2.40
201617_x_at	*CALD1*	Caldesmon 1	2.0887E-05	2.27
201645_at	*TNC*	Tenascin C	5.77047E-06	2.80
201792_at	*AEBP1*	AE binding protein 1	8.37051E-07	2.26
201842_s_at	*EFEMP1*	EGF-containing fibulin-like extracellular matrix protein 1	1.49905E-05	2.29
201843_s_at	*EFEMP1*	EGF-containing fibulin-like extracellular matrix protein 1	1.47168E-05	2.26
202133_at	*WWTR1*	WW domain containing transcription regulator 1	7.44281E-07	2.47
202207_at	*ARL4C*	ADP-ribosylation factor-like 4C	6.6529E-09	2.25
202222_s_at	*DES*	Desmin	1.79349E-06	3.31
202237_at	*NNMT*	Nicotinamide *N*-methyltransferase	5.67217E-07	2.66
202238_s_at	*NNMT*	Nicotinamide *N*-methyltransferase	2.18919E-07	2.46
202274_at	*ACTG2*	Actin, gamma 2, smooth muscle, enteric	4.68424E-07	3.52
202291_s_at	*MGP*	Matrix GLA protein	6.19879E-07	3.62

Probe ID	Gene	Protein	P-value	Proportional change
202310_s_at	*COL1A1*	Collagen, type I, alpha 1	0.000563428	2.20
202311_s_at	*COL1A1*	Collagen, type I, alpha 1	0.000616479	2.23
202363_at	*SPOCK1*	Sparc/osteonectin, cwcv and kazal-like domains proteoglycan (testican)	4.23499E-08	2.97
202403_s_at	*COL1A2*	Collagen, type I, alpha 2	6.92721E-05	2.05
202404_s_at	*COL1A2*	Collagen, type I, alpha 2	0.000215944	2.03
202437_s_at	*CYP1B1*	Cytochrome P450, family 1, subfamily B, polypeptide 1	7.75893E-06	3.21
202498_s_at	*SLC2A3*	Solute carrier family 2 (facilitated glucose transporter), member 3	5.8174E-06	2.26
202499_s_at	*SLC2A3*	Solute carrier family 2 (facilitated glucose transporter), member 3	4.31837E-06	2.70
202555_s_at	*MYLK*	Myosin light chain kinase	2.82138E-06	2.58
202628_s_at	*SERPINE1*	Serpin peptidase inhibitor, clade E (nexin, plasminogen activator)	0.000116651	2.03
202766_s_at	*FBN1*	Fibrillin 1	1.74402E-06	2.35
202988_s_at	*RGS1*	Regulator of G-protein signaling 1	7.61767E-12	3.77
203083_at	*THBS2*	Thrombospondin 2	1.97222E-06	3.01
203381_s_at	*APOE*	Apolipoprotein E	6.47221E-06	2.02
203951_at	*CNN1*	Aalponin 1, basic, smooth muscle	5.34586E-07	3.30
204030_s_at	*IQCJ-SCHIP1*	IQ motif containing J-schwannomin interacting protein 1 read-	3.253E-08	2.21
	/// SCHIP1	/// schwannomin interacting protein 1		
204051_s_at	*SFRP4*	Secreted frizzled-related protein 4	8.23252E-10	4.55
204052_s_at	*SFRP4*	Secreted frizzled-related protein 4	9.92564E-10	3.99
204069_at	*MEIS1*	Meis homeobox 1	2.8713E-06	2.15
204083_s_at	*TPM2*	Tropomyosin 2 (beta)	1.22293E-06	2.71
204135_at	*FILIP1L*	Filamin A interacting protein 1-like	1.90475E-06	2.01
204457_s_at	*GAS1*	Growth arrest-specific 1	1.60279E-05	3.75
204472_at	*GEM*	GTP binding protein overexpressed in skeletal muscle	6.16482E-11	2.98
204619_s_at	*VCAN*	Versican	1.80097E-05	2.21
204620_s_at	*VCAN*	Versican	3.28707E-05	2.17
204622_x_at	*NR4A2*	Nuclear receptor subfamily 4, group A, member 2	4.15508E-05	2.11
204894_s_at	*AOC3*	Amine oxidase, copper containing 3 (vascular adhesion protein 1)	4.01865E-05	2.10
204938_s_at	*PLN*	Phospholamban	4.08198E-06	3.30
204939_s_at	*PLN*	Phospholamban	1.19057E-06	3.38
204940_at	*PLN*	Phospholamban	2.58764E-06	3.49
205422_s_at	*ITGBL1*	Integrin, beta-like 1 (with EGF-like repeat domains)	1.09685E-07	3.62
205525_at	*CALD1*	Caldesmon 1	3.18885E-06	2.08
205547_s_at	*TAGLN*	Transgelin	1.20093E-06	3.12
205549_at	*PCP4*	Purkinje cell protein 4	0.001902231	2.27
205713_s_at	*COMP*	Cartilage oligomeric matrix protein	2.80629E-09	2.83
205767_at	*EREG*	Epiregulin	0.015105334	2.02
205880_at	*PRKD1*	Protein kinase D1	4.49555E-07	2.02
205941_s_at	*COL10A1*	Collagen, type X, alpha 1	1.88032E-06	2.85
206025_s_at	*TNFAIP6*	Tumor necrosis factor, alpha-induced protein 6	0.001039539	2.00

Probe ID	Gene	Protein	P-value	Proportional change
206211_at	*SELE*	Selectin E	1.84189E-05	2.24
206224_at	*CST1*	Cystatin SN	0.000510955	2.54
206552_s_at	*TAC1*	Tachykinin, precursor 1	0.009523889	2.15
206577_at	*VIP*	Vasoactive intestinal peptide	0.000487317	2.57
207173_x_at	*CDH11*	Cadherin 11, type 2, OB-cadherin (osteoblast)	1.13853E-06	2.33
207191_s_at	*ISLR*	Immunoglobulin superfamily containing leucine-rich repeat	5.93722E-08	2.17
208078_s_at	*SIK1*	Salt-inducible kinase 1	5.33998E-08	2.02
208131_s_at	*PTGIS*	Prostaglandin I2 (prostacyclin) synthase	1.09137E-06	2.37
208747_s_at	*C1S*	Complement component 1, s subcomponent	6.15078E-06	2.03
209101_at	*CTGF*	Connective tissue growth factor	1.954E-07	2.36
209209_s_at	*FERMT2*	Fermitin family member 2	5.19974E-06	2.09
209210_s_at	*FERMT2*	Fermitin family member 2	5.32763E-06	2.24
209335_at	*DCN*	Decorin	0.000271945	2.11
209469_at	*GPM6A*	Glycoprotein M6A	1.13583E-05	2.76
209470_s_at	*GPM6A*	Glycoprotein M6A	2.60505E-05	2.41
209656_s_at	*TMEM47*	Transmembrane protein 47	2.33312E-05	2.04
209763_at	*CHRDL1*	Chordin-like 1	1.87586E-05	2.10
209875_s_at	*SPP1*	Secreted phosphoprotein 1	1.10445E-05	3.86
210004_at	*OLR1*	Oxidized low density lipoprotein (lectin-like) receptor 1	1.40651E-06	2.56
210163_at	*CXCL11*	Chemokine (C-X-C motif) ligand 11	0.00413012	2.22
210170_at	*PDLIM3*	PDZ and LIM domain 3	3.57639E-06	2.20
210299_s_at	*FHL1*	Four and a half LIM domains 1	0.000496099	2.08
210302_s_at	*MAB21L2*	Mab-21-like 2 (C. elegans)	1.37063E-05	2.58
210495_x_at	*FN1*	Fibronectin 1	0.000177182	2.17
210511_s_at	*INHBA*	Inhibin, beta A	1.15757E-06	2.64
210517_s_at	*AKAP12*	A kinase (PRKA) anchor protein 12	1.34672E-06	2.32
210764_s_at	*CYR61*	Cysteine-rich, angiogenic inducer, 61	3.77349E-08	2.82
210809_s_at	*POSTN*	Periostin, osteoblast specific factor	0.004350075	2.01
211122_s_at	*CXCL11*	Chemokine (C-X-C motif) ligand 11	0.002741151	2.36
211571_s_at	*VCAN*	Versican	1.69458E-05	2.18
211597_s_at	*HOPX*	HOP homeobox	3.29961E-06	2.70
211719_x_at	*FN1*	Fibronectin 1	0.000199495	2.19
211896_s_at	*DCN*	Decorin	0.000239747	2.06
212077_at	*CALD1*	Caldesmon 1	1.52964E-05	2.03
212158_at	*SDC2*	Syndecan 2	3.17792E-06	2.02
212344_at	*SULF1*	Sulfatase 1	6.40288E-08	2.68
212353_at	*SULF1*	Sulfatase 1	1.53454E-07	3.21
212354_at	*SULF1*	Sulfatase 1	4.32709E-08	2.98
212464_s_at	*FN1*	Fibronectin 1	0.000150308	2.29
212489_at	*COL5A1*	Collagen, type V, alpha 1	7.30323E-05	2.00
212667_at	*SPARC*	Secreted protein, acidic, cysteine-rich (osteonectin)	1.81985E-05	2.10

Probe ID	Gene	Protein	P-value	Proportional change
212730_at	*SYNM*	Synemin, intermediate filament protein	1.15097E-06	3.38
212764_at	*ZEB1*	Zinc finger E-box binding homeobox 1	2.6995E-06	2.12
212992_at	*AHNAK2*	AHNAK nucleoprotein 2	4.32502E-05	2.16
213068_at	*DPT*	Dermatopontin	2.8673E-05	2.03
213125_at	*OLFML2B*	Olfactomedin-like 2B	2.52307E-06	2.07
213413_at	*STON1*	Stonin 1	1.97195E-06	2.01
213746_s_at	*FLNA*	Filamin A, alpha	3.12413E-06	2.10
213790_at	*ADAM12*	ADAM metallopeptidase domain 12	2.35778E-05	2.08
213905_x_at	*BGN*	Biglycan	1.46184E-05	2.31
213943_at	*TWIST1*	Twist homolog 1 (Drosophila)	2.0937E-06	2.62
214027_x_at	*DES /// FAM48A*	Desmin /// family with sequence similarity 48, member A	3.58011E-06	2.08
215446_s_at	*LOX*	Lysyl oxidase	0.00013411	2.14
215646_s_at	*VCAN*	Versican	4.95198E-05	2.43
216005_at	*TNC*	Tenascin C	4.21167E-07	3.00
216248_s_at	*NR4A2*	Nuclear receptor subfamily 4, group A, member 2	3.34829E-05	2.25
216442_x_at	*FN1*	Fibronectin 1	0.000155093	2.18
216834_at	*RGS1*	regulator of G-protein signaling 1	5.20161E-12	3.38
217428_s_at	*COL10A1*	collagen, type X, alpha 1	2.1723E-06	4.79
217762_s_at	*RAB31*	RAB31, member RAS oncogene family	3.89597E-06	2.22
217763_s_at	*RAB31*	RAB31, member RAS oncogene family	2.27983E-06	2.23
217764_s_at	*RAB31*	RAB31, member RAS oncogene family	2.67713E-06	2.31
217767_at	*C3*	Complement component 3	3.48531E-05	2.04
217967_s_at	*FAM129A*	Family with sequence similarity 129, member A	8.2629E-05	2.02
218087_s_at	*SORBS1*	Sorbin and SH3 domain containing 1	0.000359092	2.17
218468_s_at	*GREM1*	Gremlin 1	6.57618E-05	2.80
218469_at	*GREM1*	Gremlin 1	3.60594E-05	2.79
219087_at	*ASPN*	Asporin	8.97706E-08	3.78
220088_at	*C5AR1*	Complement component 5a receptor 1	1.72055E-05	2.16
221667_s_at	*HSPB8*	Heat shock 22kDa protein 8	2.31999E-06	2.55
221729_at	*COL5A2*	Collagen, type V, alpha 2	5.86389E-05	2.13
221730_at	*COL5A2*	Collagen, type V, alpha 2	3.28293E-05	2.29
221731_x_at	*VCAN*	Versican	2.21485E-05	2.14
221748_s_at	*TNS1*	Tensin 1	2.42111E-06	2.80
222088_s_at	*SLC2A14 /// SLC2A3*	Solute carrier family 2 (facilitated glucose transporter), member 14 /// solute carrier family 2 (facilitated glucose transporter), member 3	7.50757E-06	2.28
222108_at	*AMIGO2*	Adhesion molecule with Ig-like domain 2	1.40265E-05	2.06
222379_at	*KCNE4*	Potassium voltage-gated channel, Isk-related family, member 4	6.92326E-07	2.14
222877_at	---	---	8.94788E-09	2.70
223121_s_at	*SFRP2*	Secreted frizzled-related protein 2	5.48205E-06	3.14
223122_s_at	*SFRP2*	Secreted frizzled-related protein 2	2.4782E-06	4.10
223475_at	*CRISPLD1*	Cysteine-rich secretory protein LCCL domain containing 1	5.93303E-08	2.39

Probe ID	Gene	Protein	P-value	Proportional change
224396_s_at	*ASPN*	Asporin	4.75282E-08	3.04
224560_at	*TIMP2*	TIMP metallopeptidase inhibitor 2	1.80999E-05	2.08
224694_at	*ANTXR1*	Anthrax toxin receptor 1	2.0635E-06	2.55
224823_at	*MYLK*	Myosin light chain kinase	2.47351E-05	2.31
225242_s_at	*CCDC80*	Coiled-coil domain containing 80	9.22593E-06	2.15
225381_at	*LOC399959*	Hypothetical LOC399959	6.77882E-07	2.93
225481_at	*FRMD6*	FERM domain containing 6	1.90451E-06	2.28
225664_at	*COL12A1*	Collagen, type XII, alpha 1	4.09385E-05	2.36
225681_at	*CTHRC1*	Collagen triple helix repeat containing 1	1.40753E-06	3.41
225688_s_at	*PHLDB2*	Pleckstrin homology-like domain, family B, member 2	2.743E-05	2.11
225710_at	*GNB4*	Guanine nucleotide binding protein (G protein), beta polypeptide 4	5.4662E-07	2.02
225720_at	*SYNPO2*	Synaptopodin 2	4.58445E-05	2.30
225762_x_at	*LOC284801*	Hypothetical protein LOC284801	1.10206E-14	3.22
225767_at	*LOC284801*	Hypothetical protein LOC284801	2.5843E-18	5.37
225782_at	*MSRB3*	Methionine sulfoxide reductase B3	1.67805E-06	2.75
225790_at	*MSRB3*	Methionine sulfoxide reductase B3	1.71053E-06	2.32
225895_at	*SYNPO2*	Synaptopodin 2	8.00071E-05	2.95
225946_at	*RASSF8*	Ras association (RalGDS/AF-6) domain family (N-terminal) member 8	7.28589E-08	2.22
226066_at	*MITF*	Microphthalmia-associated transcription factor	6.72249E-07	2.18
226103_at	*NEXN*	Nexilin (F actin binding protein)	1.96854E-06	2.62
226237_at	*COL8A1*	Collagen, type VIII, alpha 1	4.10517E-07	3.44
226303_at	*PGM5*	Phosphoglucomutase 5	7.85843E-05	2.21
226311_at	*ADAMTS2*	ADAM metallopeptidase with thrombospondin type 1 motif, 2	4.69123E-05	2.18
226517_at	*BCAT1*	Branched chain amino-acid transaminase 1, cytosolic	2.52937E-05	2.06
226545_at	*CD109*	CD109 molecule	2.82547E-05	2.16
226677_at	*ZNF521*	Zinc finger protein 521	7.80947E-07	2.09
226777_at	*ADAM12*	ADAM metallopeptidase domain 12	3.81694E-05	2.54
226834_at	---	---	6.25067E-05	2.03
226930_at	*FNDC1*	Fibronectin type III domain containing 1	1.42194E-08	3.73
227061_at	*LOC100506621*	Hypothetical LOC100506621	1.38888E-05	2.15
227099_s_at	*C11orf96*	Chromosome 11 open reading frame 96	2.4922E-08	2.90
227140_at	*INHBA*	Inhibin, beta A	4.82673E-06	3.22
227235_at	*GUCY1A3*	Guanylate cyclase 1, soluble, alpha 3	9.90574E-07	2.68
227236_at	*TSPAN2*	Tetraspanin 2	1.15802E-07	2.14
227399_at	*VGLL3*	Vestigial like 3 (*Drosophila*)	2.88028E-05	2.37
227529_s_at	*AKAP12*	A kinase (PRKA) anchor protein 12	1.63519E-06	2.38
227566_at	*NTM*	Neurotrimin	3.275E-07	2.71
227623_at	*CACNA2D1*	Calcium channel, voltage-dependent, alpha 2/delta subunit 1	1.48825E-06	2.29
227662_at	*SYNPO2*	Synaptopodin 2	0.000101105	2.92
227697_at	*SOCS3*	Suppressor of cytokine signaling 3	5.55613E-05	2.40
227826_s_at	---	---	8.06379E-06	3.36

Probe ID	Gene	Protein	P-value	Proportional change
227827_at	---	---	4.25676E-06	3.76
228133_s_at	*MYH11*	Myosin, heavy chain 11, smooth muscle	2.56294E-05	2.60
228186_s_at	*RSPO3*	R-spondin 3 homolog (*Xenopus laevis*)	4.02828E-06	2.01
228202_at	*PLN*	Phospholamban	1.90113E-06	3.93
228640_at	*PCDH7*	Protocadherin 7	7.0205E-06	2.50
229218_at	*COL1A2*	Collagen, type I, alpha 2	9.85169E-06	2.08
229271_x_at	*COL11A1*	Collagen, type XI, alpha 1	1.03103E-05	2.32
229530_at	*GUCY1A3*	Guanylate cyclase 1, soluble, alpha 3	1.16037E-06	2.25
229554_at	---	---	1.15598E-05	2.32
229802_at	---	---	3.6632E-05	2.37
230493_at	*SHISA2*	Shisa homolog 2 (*Xenopus laevis*)	1.7547E-06	2.18
230746_s_at	*LOC100288985*	Hypothetical protein LOC100288985	2.98305E-07	2.10
231579_s_at	*TIMP2*	TIMP metallopeptidase inhibitor 2	2.79382E-05	2.07
231766_s_at	*COL12A1*	Collagen, type XII, alpha 1	6.96345E-05	2.43
231879_at	*COL12A1*	Collagen, type XII, alpha 1	5.57201E-05	2.38
232113_at	---	---	2.88882E-07	2.62
232298_at	*LOC401093*	Hypothetical LOC401093	1.32384E-05	2.12
235183_at	---	---	5.11894E-06	2.14
235944_at	*HMCN1*	Hemicentin 1	2.89194E-07	2.50
236179_at	*CDH11*	Cadherin 11, type 2, OB-cadherin (osteoblast)	2.58445E-06	2.56
236297_at	---	---	1.96659E-07	2.21
238481_at	*MGP*	Matrix GLA protein	4.22456E-08	3.17
238623_at	---	---	1.99645E-08	2.74
32128_at	*CCL18*	Chemokine (C-C motif) ligand 18 (pulmonary and activation-regulated)	8.52833E-05	2.53
37512_at	*HSD17B6*	Hydroxysteroid (17-beta) dehydrogenase 6 homolog (mouse)	6.80377E-06	2.28
37892_at	*COL11A1*	Collagen, type XI, alpha 1	9.41527E-07	3.34
1552365_at	*SCIN*	Scinderin	0.001827943	-2.11
1552502_s_at	*RHBDL2*	Rhomboid, veinlet-like 2 (*Drosophila*)	2.55739E-06	-2.14
1553828_at	*FAM55A*	Family with sequence similarity 55, member A	2.91387E-05	-2.48
1554436_a_at	*REG4*	Regenerating islet-derived family, member 4	0.000212951	-5.23
1555962_at	*B3GNT7*	UDP-GlcNAc:betaGal beta-1,3-N-acetylglucosaminyltransferase 7	7.154E-05	-3.04
1555963_x_at	*B3GNT7*	UDP-GlcNAc:betaGal beta-1,3-N-acetylglucosaminyltransferase 7	9.15242E-05	-3.38
203240_at	*FCGBP*	Fc fragment of IgG binding protein	4.78956E-05	-4.30
203559_s_at	*ABP1*	Amiloride binding protein 1 [amine oxidase (copper-containing)]	7.27109E-07	-2.20
203649_s_at	*PLA2G2A*	Phospholipase A2, group IIA (platelets, synovial fluid)	0.002158679	-2.46
203691_at	*PI3*	Peptidase inhibitor 3, skin-derived	2.39096E-05	-2.54
203908_at	*SLC4A4*	Solute carrier family 4, sodium bicarbonate cotransporter, member 4	1.09103E-05	-4.34
204130_at	*HSD11B2*	Hydroxysteroid (11-beta) dehydrogenase 2	3.28616E-06	-2.02
204213_at	*PIGR*	Polymeric immunoglobulin receptor	3.18471E-05	-3.53
204673_at	*MUC2*	Mucin 2, oligomeric mucus/gel-forming	0.000154428	-3.61

Probe ID	Gene	Protein	P-value	Proportional change
204818_at	*HSD17B2*	Hydroxysteroid (17-beta) dehydrogenase 2	1.61903E-05	-2.56
204895_x_at	*MUC4*	Mucin 4, cell surface associated	4.95801E-05	-2.17
205097_at	*SLC26A2*	Solute carrier family 26 (sulfate transporter), member 2	0.007081477	-2.15
205185_at	*SPINK5*	Serine peptidase inhibitor, Kazal type 5	1.51319E-05	-2.36
205259_at	*NR3C2*	Nuclear receptor subfamily 3, group C, member 2	0.000364922	-2.02
205825_at	*PCSK1*	Proprotein convertase subtilisin/kexin type 1	0.016576148	-2.16
205950_s_at	*CA1*	Carbonic anhydrase I	1.42429E-06	-4.31
205979_at	*SCGB2A1*	Secretoglobin, family 2A, member 1	2.41977E-05	-3.04
206000_at	*MEP1A*	Meprin A, alpha (PABA peptide hydrolase)	0.001001283	-2.52
206143_at	*SLC26A3*	Solute carrier family 26, member 3	0.00055994	-4.58
206198_s_at	*CEACAM7*	Carcinoembryonic antigen-related cell adhesion molecule 7	0.000346086	-3.98
206199_at	*CEACAM7*	Carcinoembryonic antigen-related cell adhesion molecule 7	0.000404387	-3.15
206208_at	*CA4*	Carbonic anhydrase IV	3.14437E-06	-2.60
206209_s_at	*CA4*	Carbonic anhydrase IV	9.1296E-07	-3.60
206262_at	*ADH1C*	Alcohol dehydrogenase 1C (class I), gamma polypeptide	1.11801E-05	-4.05
206268_at	*LEFTY1*	Left-right determination factor 1	0.006935695	-2.48
206561_s_at	*AKR1B10*	Aldo-keto reductase family 1, member B10 (aldose reductase)	0.000110476	-2.30
206641_at	*TNFRSF17*	Tumor necrosis factor receptor superfamily, member 17	6.65093E-05	-2.02
206664_at	*SI*	Sucrase-isomaltase (alpha-glucosidase)	2.842E-07	-8.14
207003_at	*GUCA2A*	Guanylate cyclase activator 2A (guanylin)	1.76864E-06	-3.34
207214_at	*SPINK4*	Serine peptidase inhibitor, Kazal type 4	2.99566E-05	-5.21
207222_at	*PLA2G10*	Phospholipase A2, group X	1.45449E-05	-2.09
207245_at	*UGT2B17*	UDP glucuronosyltransferase 2 family, polypeptide B17	0.002842665	-3.42
208450_at	*LGALS2*	Lectin, galactoside-binding, soluble, 2	4.05288E-06	-2.79
209116_x_at	*HBB*	Hemoglobin, beta	2.37634E-05	-2.60
209301_at	*CA2*	Carbonic anhydrase II	0.000136418	-3.05
209458_x_at	*HBA1 /// HBA2*	Hemoglobin, alpha 1 /// hemoglobin, alpha 2	0.000257361	-2.06
209613_s_at	*ADH1B*	Alcohol dehydrogenase 1B (class I), beta polypeptide	0.016020505	-2.04
209752_at	*REG1A*	Regenerating islet-derived 1 alpha	0.042615985	-2.28
210107_at	*CLCA1*	Chloride channel accessory 1	9.72618E-07	-7.23
210174_at	*NR5A2*	Nuclear receptor subfamily 5, group A, member 2	2.06347E-05	-2.02
210738_s_at	*SLC4A4*	Solute carrier family 4, sodium bicarbonate cotransporter, member 4	7.18965E-05	-2.30
211696_x_at	*HBB*	Hemoglobin, beta	1.12554E-05	-2.39
211699_x_at	*HBA1 /// HBA2*	Hemoglobin, alpha 1 /// hemoglobin, alpha 2	0.000153371	-2.02
211745_x_at	*HBA1 /// HBA2*	Hemoglobin, alpha 1 /// hemoglobin, alpha 2	0.000163306	-2.12
211848_s_at	*CEACAM7*	Carcinoembryonic antigen-related cell adhesion molecule 7	0.000349566	-3.17
212531_at	*LCN2*	Lipocalin 2	5.05852E-05	-2.77
212592_at	*IGJ*	Immunoglobulin J polypeptide, linker protein for immunoglobulin alpha and mu polypeptides	4.97675E-05	-4.06
212768_s_at	*OLFM4*	Olfactomedin 4	7.64335E-05	-6.94
212814_at	*AHCYL2*	Adenosylhomocysteinase-like 2	5.00094E-07	-2.14
214142_at	*ZG16*	Zymogen granule protein 16 homolog (rat)	6.0815E-07	-7.33

Probe ID	Gene	Protein	P-value	Proportional change
214414_x_at	*HBA1 /// HBA2*	Hemoglobin, alpha 1 /// hemoglobin, alpha 2	4.54995E-05	-2.49
214433_s_at	*SELENBP1*	Selenium binding protein 1	4.2296E-05	-2.33
214598_at	*CLDN8*	Claudin 8	1.80976E-06	-4.81
215657_at	*SLC26A3*	Solute carrier family 26, member 3	0.002370526	-2.26
217022_s_at	*IGHA1 /// IGHA2*	Immunoglobulin heavy constant alpha 1 /// immunoglobulin heavy constant alpha 2 (A2m marker)	2.43669E-05	-3.50
	LOC100126583	/// hypothetical LOC100126583		
217109_at	*MUC4*	Mucin 4, cell surface associated	1.35267E-05	-3.86
217110_s_at	*MUC4*	Mucin 4, cell surface associated	3.51375E-05	-3.27
217232_x_at	*HBB*	Hemoglobin, beta	2.05507E-05	-2.09
217238_s_at	*ALDOB*	Aldolase B, fructose-bisphosphate	0.00142396	-2.04
217414_x_at	*HBA1 /// HBA2*	Hemoglobin, alpha 1 /// hemoglobin, alpha 2	0.000140008	-2.10
217546_at	*MT1M*	Metallothionein 1M	0.000408888	-2.82
219014_at	*PLAC8*	Placenta-specific 8	0.001224132	-2.25
219727_at	*DUOX2*	Dual oxidase 2	1.335E-05	-3.53
219795_at	*SLC6A14*	Solute carrier family 6 (amino acid transporter), member 14	0.000316091	-3.36
219948_x_at	*UGT2A3*	UDP glucuronosyltransferase 2 family, polypeptide A3	3.91209E-05	-4.09
220026_at	*CLCA4*	Chloride channel accessory 4	6.50973E-07	-6.86
220030_at	*STYK1*	Serine/threonine/tyrosine kinase 1	3.96282E-06	-2.35
220075_s_at	*CDHR5*	Cadherin-related family member 5	2.10493E-05	-2.43
220376_at	*LRRC19*	Leucine rich repeat containing 19	0.000233493	-2.74
220645_at	*FAM55D*	Family with sequence similarity 55, member D	6.0041E-05	-2.21
220812_s_at	*HHLA2*	HERV-H LTR-associating 2	1.4047E-06	-2.09
220834_at	*MS4A12*	Membrane-spanning 4-domains, subfamily A, member 12	1.79419E-06	-5.32
221004_s_at	*ITM2C*	Integral membrane protein 2C	2.84249E-05	-2.08
223447_at	*REG4*	Regenerating islet-derived family, member 4	0.000189189	-4.21
223484_at	*C15orf48*	Chromosome 15 open reading frame 48	1.34775E-05	-2.09
223551_at	*PKIB*	Protein kinase (cAMP-dependent, catalytic) inhibitor beta	0.001012167	-2.33
223597_at	*ITLN1*	Intelectin 1 (galactofuranose binding)	1.9742E-07	-8.23
223952_x_at	*DHRS9*	Dehydrogenase/reductase (SDR family) member 9	0.000600741	-2.08
224009_x_at	*DHRS9*	Dehydrogenase/reductase (SDR family) member 9	0.000308104	-2.35
224027_at	*CCL28*	Chemokine (C-C motif) ligand 28	6.04109E-07	-2.63
224412_s_at	*TRPM6*	Transient receptor potential cation channel, subfamily M, member 6	0.000220103	-2.47
226147_s_at	*PIGR*	Polymeric immunoglobulin receptor	0.00063626	-3.95
226654_at	*MUC12*	Mucin 12, cell surface associated	0.001674357	-2.36
226974_at	*NEDD4L*	Neural precursor cell expressed, developmentally down-regulated 4-like	4.62046E-06	-2.10
227048_at	*LAMA1*	Laminin, alpha 1	4.34789E-05	-2.16
227725_at	*ST6GALNAC1*	ST6 (alpha-N-acetyl-neuraminyl-2,3-beta-galactosyl-1,3)	0.000642755	-2.41
227735_s_at	*C10orf99*	Chromosome 10 open reading frame 99	0.001153096	-2.28
227736_at	*C10orf99*	Chromosome 10 open reading frame 99	0.003108923	-2.40
228232_s_at	*VSIG2*	V-set and immunoglobulin domain containing 2	4.10444E-06	-3.08
228241_at	*AGR3*	Anterior gradient homolog 3 (*Xenopus laevis*)	0.00398137	-2.60

Probe ID	Gene	Protein	P-value	Proportional change
229070_at	*C6orf105*	Chromosome 6 open reading frame 105	2.99495E-07	-3.95
229254_at	*MFSD4*	Major facilitator superfamily domain containing 4	6.75206E-05	-2.83
229831_at	*CNTN3*	Contactin 3 (plasmacytoma associated)	0.000235636	-2.11
234632_x_at	---	---	3.1752E-05	-3.17
237530_at	---	---	4.31807E-05	-2.03
238143_at	*LOC646627*	Phospholipase inhibitor	0.000458742	-2.28
238750_at	*CCL28*	Chemokine (C-C motif) ligand 28	1.24326E-06	-2.94
238846_at	*TNFRSF11A*	Tumor necrosis factor receptor superfamily, member 11a, NFKB activator	0.000256856	-2.14
239370_at	*LOC100505633*	Hypothetical LOC100505633	6.16647E-06	-2.10
239673_at	---	---	6.3915E-06	-2.47
239994_at	---	---	1.10317E-05	-2.10
240856_at	*GPR120*	G protein-coupled receptor 120	0.000388776	-2.03
241994_at	*XDH*	Xanthine dehydrogenase	5.03297E-06	-2.32
242601_at	*HEPACAM2*	HEPACAM family member 2	1.47154E-05	-5.35
41469_at	*PI3*	Peptidase inhibitor 3, skin-derived	1.24208E-05	-2.46

Supplementary Table 5: Distribution of partitions derived from hierarchical clustering of patients with primary colorectal carcinoma across different timepoints: pre, before hepatic pedicle clamping; post, after clamping; 10’, 10 minutes after resection; 20’, 20 minutes after resection; and 45’, 45 minutes after resection.

Partition	**Normal tissue**<br>Patient no.	**Tumor tissue**<br>Patient no.
Pre/10’ 20’ 45’	2, 3, 4, 7, 8, 9, 10, 12, 13, 15, 16, 17, 18, 21, 22, 23, 25, 26, 27, 28, 30, 32, 33, 34, 35, 36, 37, 38, 39, 41, 42, 43, 46, 47, 48	2, 3, 4, 9, 10, 11, 12, 16, 17, 20, 21, 23, 26, 27, 29, 30, 32, 34, 36, 39, 40, 42
Pre 10’/20’ 45’	11	7, 18, 43
Pre 20’/10’ 45’		33, 41
Pre 45’/10’ 20’	40	35, 46, 48
10’/pre 20’ 45’	5	22
20’/pre 10’ 45’	20	5, 37, 28
45’/pre 10’ 20’	29	8, 13, 15, 25, 38, 47
